# Anti‐Acidification and Immune Regulation by Nano‐Ceria‐Loaded Mg–Al Layered Double Hydroxide for Rheumatoid Arthritis Therapy

**DOI:** 10.1002/advs.202307094

**Published:** 2023-12-08

**Authors:** Hao Fu, Yuedong Guo, Wenming Fang, Jiaxing Wang, Ping Hu, Jianlin Shi

**Affiliations:** ^1^ Shanghai Institute of Ceramics Chinese Academy of Sciences Research Unit of Nanocatalytic Medicine in Specific Therapy for Serious Disease Chinese Academy of Medical Sciences (2021RU012) Shanghai 200050 P. R. China; ^2^ Platform of Nanomedicine Translation Shanghai Tenth People's Hospital Medical School of Tongji University 38 Yun‐xin Road Shanghai 200435 P. R. China; ^3^ Department of Orthopaedics Shanghai Jiao Tong University Affiliated Sixth People's Hospital Shanghai Jiao Tong University Shanghai 200233 P. R. China

**Keywords:** acid neutralization, enhanced osteogenesis, immune modulation, rheumatoid arthritis (RA), ROS scavenging

## Abstract

Rheumatoid arthritis (RA) is a chronic autoimmune disease featuring an abnormal immune microenvironment and resultant accumulation of hydrogen ions (H^+^) produced by activated osteoclasts (OCs). Currently, clinic RA therapy can hardly achieve sustained or efficient therapeutic outcomes due to the failures in generating sufficient immune modulation and manipulating the accumulation of H^+^ that deteriorates bone damage. Herein, a highly effective immune modulatory nanocatalytic platform, nanoceria‐loaded magnesium aluminum layered double hydroxide (LDH‐CeO_2_), is proposed for enhanced immune modulation based on acid neutralization and metal ion inherent bioactivity. Specifically, the mild alkaline LDH initiates significant M2 repolarization of macrophages triggered by the elevated antioxidation effect of CeO_2_ via neutralizing excessive H^+^ in RA microenvironment, thus resulting in the efficient recruitment of regulatory T cell (Treg) and suppressions on T helper 17 cell (Th 17) and plasma cells. Moreover, the osteogenic activity is stimulated by the Mg ion released from LDH, thereby promoting the damaged bone healing. The encouraging therapeutic outcomes in adjuvant‐induced RA model mice demonstrate the high feasibility of such a therapeutic concept, which provides a novel and efficient RA therapeutic modality by the immune modulatory and bone‐repairing effects of inorganic nanocatalytic material.

## Graphical abstract

The study proposes a novel strategy for rheumatoid arthritis (RA) immune therapy by an LDH‐CeO_2_ nanocatalytic platform, featuring efficiently enhanced antioxidation activity of CeO_2_ NPs at the acidic RA lesions by anti‐acidification and the resultant in situ anti‐inflammatory modulation. This nanoplatform, in synergy with promoted osteogenesis by Mg^2+^, generates a satisfactory RA mitigation, thus presenting a promising prospect for clinical RA treatment.

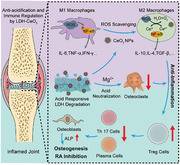

## 1 Introduction

As a chronic progressive autoimmune inflammatory disease, rheumatoid arthritis (RA) is featured by typical inflammatory symptoms.^[^ ^1^ ^]^ Presently, RA accounts for ≈1% of global prevalence, and patients with advanced RA suffer from severe pains and physical inconvenience of joints, which greatly deteriorates the quality of life.^[^ ^1^, ^2^ ^]^ Accordingly, the investigations on developing therapeutics against RA is of great significance.

RA originates from complex interplays which still remain largely unclear.^[^ ^1^, ^3^ ^]^ However, researchers have demonstrated that abnormal immune balance is strongly associated with the pathogenesis of RA.^[^ ^4^ ^]^ The overproduction of reactive oxygen species (ROS) is an important cause that leads to oxidative damage to articular cells and the accumulation of proinflammatory immune cells (like M1 macrophages).^[^ ^1^, ^5^ ^]^ The prevalence of proinflammatory M1 macrophages induced by ROS secrete abundant inflammatory cytokines like interleukin‐6 (IL‐6) or tumor necrosis factor α (TNF‐α), triggering the apoptosis of osteoblasts and activation of osteoclasts (OCs).^[^ ^4^ ^]^ Moreover, the inflammatory immune microenvironment in joints is then aggravated by Th 17 or plasma cells recruited by inflammatory cytokines.^[^ ^4^ ^]^ Besides, the excessive H^+^ secreted by activated OCs promotes bone destruction and RA progression.^[^ ^5^, ^6^ ^]^ Resultantly, inhibiting the inflammatory microenvironment by ROS scavenging is a proper RA treatment method.^[^ ^7^ ^]^ In diseases associated with high oxidative stress, ceria oxide nanoparticles (denoted as CeO_2_ NPs) have been widely investigated for their multi‐enzyme‐like activities mimicking such as superoxide dismutase (SOD) and catalase (CAT), etc.^[^ ^8^ ^]^ Under neutral or physical condition, redox reactions are initiated between CeO_2_ NPs and H_2_O_2_ (Equations (1)–(3)) or O_2_•^−^ (Equations (4) and (5)).^[^ ^8^, ^9^ ^]^

(1) $$H_{2}O_{2}+{\mathrm{Ce}}^{3+}+H^{+}\to {\mathrm{Ce}}^{4+}+•\mathrm{OH}+H_{2}O$$

(2) $$•\mathrm{OH}+H_{2}O_{2}\to {\mathrm{HO}}_{2}^{-}+H_{2}O$$

(3) $${\mathrm{HO}}_{2}^{-}+{\mathrm{Ce}}^{4+}\to O_{2}+H^{+}+{\mathrm{Ce}}^{3+}$$

(4) $$O_{2}^{•-}+{\mathrm{Ce}}^{4+}\to O_{2}+{\mathrm{Ce}}^{3+}$$

(5) $$O_{2}^{•-}+{\mathrm{Ce}}^{3+}+2H^{+}\to H_{2}O_{2}+{\mathrm{Ce}}^{4+}$$

However, when subjecting to acidic condition (RA for example), excessive hydrogen ions (H^+^) will prevent the conversion of Ce^4+^ to Ce^3+^, deteriorating the antioxidation effect of CeO_2_ NPs.^[^ ^8^, ^10^ ^]^ Resultantly, CeO_2_ NPs may generate reluctant antioxidative efficacy in RA, which is accompanied by extremely acidic environment (pH ≈5‐6) resulting from abundant H^+^ secreted by activated osteoclasts.^[^ ^5^, ^6^ ^]^ Therefore, to enhance the ROS scavenging activity of CeO_2_ NPs, we conceive that a facile acid−base neutralization strategy will help.

Currently, first‐line drugs, disease‐modifying anti‐rheumatic drugs (DMARDS), nonsteroidal anti‐inflammatory drugs (NSAIDs), biological targeting DMARDS, and corticosteroids, have been widely applied against RA.^[^ ^11^ ^]^ However, the severe side effects and unsatisfactory therapeutic results have hindered their long‐term uses.^[^ ^12^ ^]^ For example, DMARDS is associated with substantial hepatotoxicity;^[^ ^12^, ^13^ ^]^ the use of NSAIDs can cause serious gastrointestinal reactions or even peptic ulcers;^[^ ^12^, ^13^ ^]^ corticosteroids, though displaying excellent curative effect in a short time scale, may arouse irreversible metabolic disorders or even immune injury frequently.^[^ ^12^, ^14^ ^]^ More importantly, current therapeutics generate unsatisfactory outcomes by ignoring the regulations of immune balance and acidity at the lesion sites, which results in the continued destruction of the joints.^[^ ^4^, ^15^ ^]^ Therefore, inducing anti‐inflammatory M2 polarization of macrophages by scavenging ROS and the subsequent anti‐inflammatory remodulation in synergy with acid neutralization may be an effective and relatively safe strategy in treating RA.

Layer double hydroxide (LDH) have attracted intensive attention for their excellent biocompatibility, pH‐responsive biodegradability, easy surface modification, and acidic neutralization.^[^ ^16^ ^]^ Clinically, MgAl‐LDH has been successfully applied in neutralizing the acidity in the stomach for relieving symptoms associated with hyperacidity.^[^ ^16a,c^ ^]^ Besides, thanks to the diversity of metal ion composition, LDH exhibits impressive immune modulatory capability based on the inherent properties of the divalent metal ions.^[^ ^16a,c^ ^]^ For example, Mg^2+^ can inhibit the formation of osteoclasts by preventing monocyte fusion and present potent anti‐inflammatory function by stimulating the M2 polarization of macrophages;^[^ ^17^ ^]^ Mg^2+^ is also efficient in activating osteoblasts and facilitates the damaged bone healing.^[^ ^18^ ^]^ In all, MgAl‐LDH may be promising in promoting the ROS scavenging of CeO_2_ NPs by acid neutralization, thus largely mitigating the inflammatory microenvironment and relieving the bone destruction at the RA site by released Mg^2+^.

**Scheme 1:**
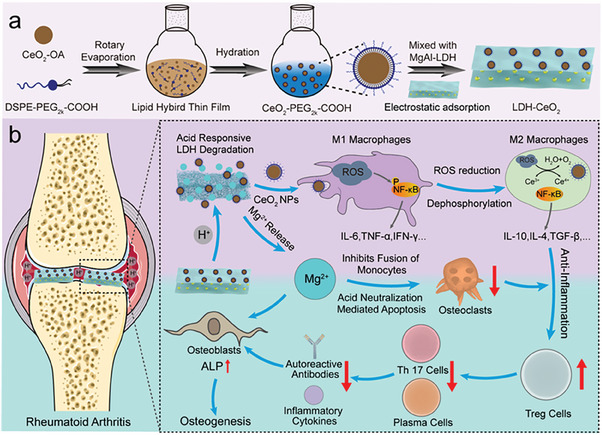
Illustration of LDH‐CeO_2_ preparation and its regulation mechanism on RA microenvironment. a) Preparation of nanocatalytic material LDH‐CeO_2_ for RA therapy. b) Mechanism of LDH‐CeO_2_ in treating RA. Briefly, the hyperacidity of RA microenvironment is neutralized by LDH, which results in the degradation of LDH releasing Mg^2+^ and CeO_2_ NPs at the lesion, and the resultant ROS scavenging activity enhancement of CeO_2_ NPs and the notable re‐polarization of M1 macrophages into M2 phenotype via dephosphorylation of nuclear transcriptional factor (NF‐κB) pathway in the meantime. Due to the abundant production of interleukin‐10 (IL‐10) and other anti‐inflammatory cytokines by M2 macrophages, the microenvironment is reshaped into anti‐inflammatory one, which lead to the accumulation of Treg cells and therefore inhibition of the inflammatory immune cells (Th 17 cells, plasma cells). Such an immune modulation in RA microenvironment then results in the enhanced osteoblast's activity and viability. Moreover, Mg^2+^ released from LDH will suppress the formation of osteoclasts by inhibiting the fusion of monocytes as well as enhance the osteogenic activity of osteoblasts, further facilitating the healing of bone erosion caused by RA.

Herein, in this work, we propose a novel approach to achieve efficient ROS scavenging and immune modulation for RA therapy based on a CeO_2_ NPs‐loaded MgAl‐LDH (LDH‐CeO_2_) nano‐platform for the first time (**Scheme** **1**). Briefly, the anti‐oxidative activity of CeO_2_ NPs is enhanced by the acid neutralization using the mild basicity of LDH after in situ injection, thus initiating the pro‐inflammatory M1 macrophage repolarization into anti‐inflammatory M2 phenotype via the dephosphorylation of nuclear transcriptional factor (NF‐κB) associated with intracellular ROS, where the re‐polarization is further strengthened by the released Mg ions from LDH. As a result, the abundantly produced M2 macrophages produce a large amount of anti‐inflammatory cytokines (such as interleukin‐10, IL‐10), which then recruits a large proportion of Treg cells, one of the most potent anti‐inflammatory immune cell cohorts, thus strongly deactivating Th 17 and plasma cells therein. Concurrently, the released Mg^2+^ further prevents the formation of osteoclasts by inhibiting the fusion of monocytes, and activates osteoblasts in the meantime, thereby suppressing bone erosion but encouraging bone healing. Such a synergistic strategy is expected to produce significantly enhanced efficacy of RA therapeutics by LDH‐CeO_2_ based on immune regulation and bone repairing enhanced by acid neutralization.

## 2 Results and Discussion

### 2.1 Synthesis and Characterization of LDH‐CeO_2_ as a Nanocatalytic Medicine

**Figure 1:**
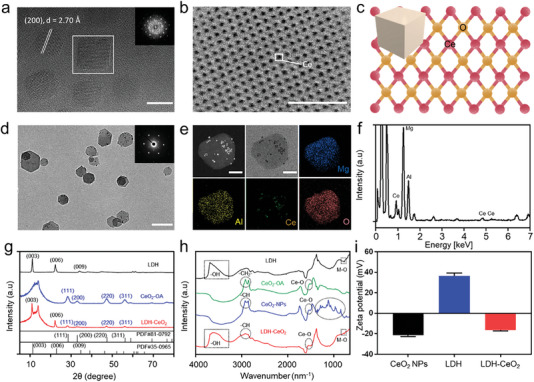
Characterization of CeO_2_ NPs, LDH and LDH‐CeO_2_. a) HRTEM and the corresponding SAED pattern and b) ABF‐STEM images of CeO_2_‐OA. Scale bar: 5 and 2 nm. c) Schematic diagram of atomic arrangement of CeO_2_‐OA NPs. d) TEM images of LDH‐CeO_2_ and corresponding SAED pattern in the inset. Scale bar: 200 nm. e) Elemental mapping images and f) EDS spectra of LDH‐CeO_2_. Scale bar: 50 nm. g) XRD patterns of LDH (black), CeO_2_‐OA (blue) and LDH‐CeO_2_ (red). h) FTIR spectra of LDH (black), CeO_2_‐OA (green), CeO_2_‐PEG‐COOH (blue) and LDH‐CeO_2_ (red). i) Zeta‐potentials of CeO_2_, LDH and LDH‐CeO_2_, and all values are presented as means ± s.d.

The anti‐oxidant CeO_2_ NPs were synthesized via a modified hydrothermal method.^[^ ^19^ ^]^ The obtained CeO_2_ modified with oleic acid (denoted as CeO_2_‐OA) was uniformly dispersed in toluene under transmission electron microscopy (TEM) observation monitoring (Figure S1a, Supporting Information) and the size distribution was 5.35 nm in average (Figure S1b, Supporting Information). High‐resolution transmission electron microscopic (HRTEM) image shows the cubic fluorite structure of cerium oxide nanocrystals with a lattice fringe distance of 2.70Ǻ attributed to the (200) plane (**Figure** **1**a), which was further confirmed by the corresponding selected‐area electron diffraction patterns (SAED) (Figure 1a).^[^ ^20^ ^]^ Annular bright‐field (ABF) scanning transmission electron microscopy (STEM) image (Figure 1b) further presents the ordered cerium atomic phase. The elemental mapping and energy‐dispersive X‐ray spectroscopy (EDS) spectra indicate the uniform distributions of Ce and O elements in CeO_2_‐OA (Figure S2a,b, Supporting Information). Moreover, the crystalline structure model of CeO_2_‐OA based on the crystallographic information file is also consistent with the above STEM results (Figure 1c). To facilitate its biomedical application, the as‐synthesized CeO_2_‐OA was modified by DSPE‐PEG_2k_‐COOH for improving their aqueous dispersion and biocompatibility.^[^ ^8^, ^9^, ^21^ ^]^ The as‐prepared PEG_2k_‐COOH modified CeO_2_ nanoparticles (CeO_2_‐PEG_2k_‐COOH, denoted as CeO_2_ NPs) exhibit excellent colloidal stability and mono‐dispersity in aqueous solution under the TEM observation (Figure S3a, Supporting Information) with an average size of ≈6.78 nm (Figure S3b, Supporting Information). In the meantime, MgAl‐LDH (LDH) materials were prepared as the carrier for CeO_2_ NPs.^[^ ^22^ ^]^ As displayed in Figure S4a (Supporting Information), the as‐prepared LDH shows excellent mono‐dispersity dispersity in aqueous phase as visulized by TEM, while the average size, calculated from TEM images, is ≈139.5 nm in lateral dimension (Figure S4b, Supporting Information). The efficient CeO_2_ NPs loading in the surface of LDH was achieved via electrostatic absorption.^[^ ^23^ ^]^ As shown in Figure 1d, the CeO_2_ NPs‐loaded LDH (denoted as LDH‐CeO_2_) has been successfully synthesized in which the CeO_2_ NPs can be clearly visulized to be attached on the surface of LDH, as also suggested by the corresponding fast Fourier transform (FFT) image showing the mixed patterns of both crystalline structures of CeO_2_ NPs and LDH.^[^ ^24^ ^]^ This TEM observation evidences the excellent dispersity of LDH‐CeO_2_ in aqueous phase. The high‐angle annular dark‐field scanning transmission electron microscopy (HAADF‐STEM) images indicate the presence of CeO_2_ NPs on the surface of LDH (Figure 1e), while the elemental mapping evidences the presence and uniform distributions of Mg, Al, Ce and O elements in the LDH‐CeO_2_ (Figure 1e), where the distribution of Ce element is clearly overlapped with that of CeO_2_ NPs observed in HAADF‐STEM. Such an elementary distribition is further confirmed by energy‐dispersive X‐ray spectroscopy (EDS) (Figure 1f). Moreover, the X‐ray diffraction (XRD) patterns verify the hexagonally crystallized structure of layered MgAl‐LDH (PDF#35‐0965), consisting of the cubic fluorite structure of CeO_2_ NPs (PDF#81‐0792) and the layered structure of LDH of the LDH‐CeO_2_ nanoplatform (Figure 1g).^[^ ^22^, ^25^ ^]^ The resultant weight percentages (Wt.%) of Mg, Al and Ce are 7.31, 2.93, and 1.42%, respectively, suggesting a Mg/Al/Ce molecule ratio of ≈26:10:1 (Table S1, Supporting Information).

To investigate the chemical state and of LDH‐CeO_2_, X‐ray photoelectron spectroscopy (XPS) was employed. The resultant fitting peak located at 1305.85 and 74.35 eV are ascribed to Mg 1s (Figure S5a, Supporting Information) and Al 2p (Figure S5b, Supporting Information), respectively, indicating the presences of Mg^2+^ and Al^3+^ in the composite materials from MgAl‐LDH.^[^ ^26^ ^]^ The Ce^3+^/Ce^4+^ ratio is 36.68/63.32%, as quantified from the fitting peaks of Ce 3d attributed to Ce^3+^ (882.7, 888.5, 898.35, 917 eV) and Ce^4+^ (885.6, 901.9 eV), which favors the antioxidation activity elevation of CeO_2_ NPs of high‐enough Ce^3+^ percentages (Figure S5c, Supporting Information).^[^ ^8b^ ^]^ Also, O 1s band could be deconvoluted into four sub‐bands as “metal‐O” (M‐O, 531.5 eV), “H‐O” (532.1 eV), “Ce‐O” (530.6 eV) and H_2_O (533.1 eV) (Figure S5d, Supporting Information), further verifying the successful preparation of LDH‐CeO_2_.^[^ ^27^ ^]^ In addition, the Fourier transform infrared (FTIR) spectra reveal the presences of absorption bands at 3630–3475 cm^−1^ and absorption peaks at 788 and 538 cm^−1^, belonging to the stretching vibrations of –OH groups and metal–OH (M–OH) in LDH, respectively, in both LDH and LDH‐CeO_2_ (Figure 1h).^[^ ^16^, ^22^ ^]^ The multiple bands ranging from 500 to 2000 cm^−1^ in CeO_2_‐NPs is attributed to the organic molecule modification of DSPE‐PEG_2k_‐COOH, indicating the successful surface decoration of CeO_2_‐OA.^[^ ^8b^ ^]^ Moreover, the absorption bands at 1500–1600 cm^−1^ of Ce─O bonds in both CeO_2_ NPs and LDH‐CeO_2_ further indicate the successful combination of LDH and CeO_2_ (Figure 1h). Besides, the zeta‐potentials of CeO_2_ NPs, LDH, and LDH‐CeO_2_ are ≈−20, +38, and −15 mV, respectively (Figure 1i), which confirms the feasible fabrication of LDH‐CeO_2_ via electrostatic interaction.^[^ ^23^ ^]^

Owing to the mild basicity, LDH is highly responsive to excessive hydrogen ions (H^+^), which make it applicable for acid neutralization.^[^ ^16a–d^ ^]^ Therefore, the time‐dependent ion release profile of LDH‐CeO_2_ in response to acidity was first investigated using ICP‐OES. As displayed in Figure S6a (Supporting Information), the Mg^2+^ release from LDH‐CeO_2_ under a relatively strong acidic condition (pH 4.5) is much faster than that under mild acidic (pH 6.5) or neutral condition (pH 7.4), guaranteeing the Mg ions supply for subsequent immune modulation or bone cell regulation in RA region. In addition, the release curves of Ce and Mg ions are analogous to each other (Figure S6b, Supporting Information). The acid neutralization effect of LDH‐CeO_2_ was further confirmed by pH value variation during LDH‐CeO_2_ addition into acidic or neutral PBS (pH 4.5, 6.5, or 7.4). As demonstrating in Figure S7a (Supporting Information), analogous to traditional PBS buffer solution (pH 7.4), LDH‐CeO_2_ could significantly prevent the acidification by titrating 1% HCl solution in comparison with pure H_2_O or CeO_2_ NPs solutions.^[^ ^16b^ ^]^ In addition, the pH value of acidic PBS solutions (pH 4.5 or 6.5) rises notably after adding LDH‐CeO_2_, which manifests the excellent acidity‐neutralization property of LDH‐CeO_2_ (Figure S7b, Supporting Information), further validating the acid neutralization capability of LDH‐CeO_2_.^[^ ^16^, ^28^ ^]^ Owing to such a specific property, this composite nanocatalytic material is applicable for regulating the hyperacidity in disease regions.

### 2.2 Promoted Antioxidative Activity under Acidic Condition by LDH‐CeO_2_

**Figure 2:**
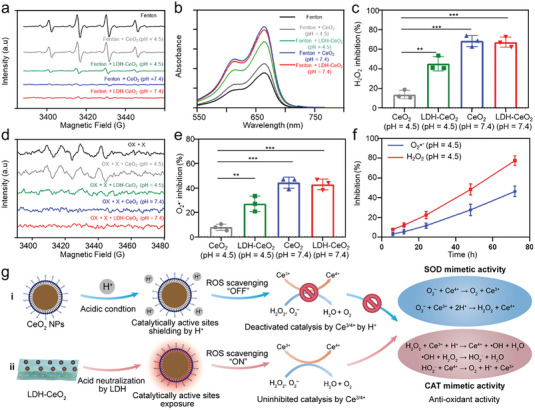
Enhanced ROS scavenging via acid neutralization by LDH‐CeO_2_. a) ESR spectra of •OH trapped by DMPO of different treatment groups. b) UV–vis absorbance spectra of MB after different treatments. c) Inhibition rate of H_2_O_2_ (CAT‐mimic activity) of different groups. d) ESR spectra of O_2_^•‐^ generated by OX + X system treated by various groups. e) Inhibition rates on O_2_^•‐^ (SOD‐mimic activity) by different groups. f) Inhibition rates on H_2_O_2_ and O_2_^•‐^ of LDH‐CeO_2_ under acidic condition for varied time periods. g) Mechanism of ROS scavenging ability of CeO_2_ NPs assisted by acid neutralization of LDH. All values are presented as means ± s.d, *n* = 3. ^*^ *P* < 0.05, ^**^ *P* < 0.01, ^***^ *P* < 0.001, ns, not significant, two‐tailed Student's *t*‐test.

CeO_2_ NPs is an anti‐oxidant with extraordinary ROS scavenging efficacy by mimicking CAT or SOD.^[^ ^9^, ^29^ ^]^ However, the antioxidation capacity of CeO_2_ NPs is much compromised under the acidic condition, which greatly hinders its application in diseases associated with hyperacidity.^[^ ^8^, ^10^ ^]^ Therefore, to guarantee the ROS scavenging efficacy, LDH is introduced to neutralize the excessive acid, thus enhancing the anti‐oxidation performance of CeO_2_ NPs. To test our hypothesis, we first examined the CAT mimic activity of LDH‐CeO_2_ in scavenging the hydroxyl radicals (•OH) or hydrogen peroxide (H_2_O_2_) by electron spin resonance (ESR) spectra.^[^ ^8^, ^30^ ^]^ As demonstrated in **Figure** **2**a, both CeO_2_ NPs and LDH‐CeO_2_ exhibits notable •OH scavenging activity under neutral condition (pH 7.4), in which the characteristic •OH pattern (1: 2: 2: 1) is weakened dramatically. However, CeO_2_ NPs became much inactive in ROS scavenging under the acidic condition (pH 4.5) because of the prevented Ce^4+^/Ce^3+^ conversion by excessive H^+^ (Figure 2a).^[^ ^8^, ^10^ ^]^ Thankfully, this phenomenon was largely reversed by the LDH‐CeO_2_ owing to the acid neutralization effect of the LDH component (Figure 2a). Furthermore, the methyl blue (MB) decolorization analysis exhibits the similar behavior as that by ESR spectroscopy, indicating the elevated anti‐oxidation capacity of CeO_2_ NPs by LDH in the acidic condition (Figure 2b).^[^ ^30^ ^]^ Also, the MB degradation percentage quantification shows the similar effect by LDH as indicated above (Figure S8, Supporting Information). In evaluating the CAT‐mimic activity, both CeO_2_ NPs and LDH‐CeO_2_ perform significant inhibitions over hydrogen peroxide (H_2_O_2_) under neutral condition. While, as comparable to the above, the inactive ROS scavenging of CeO_2_ NPs in under acidic environment can be largely reversed by the LDH‐CeO_2_ (Figure 2c). The O_2_^•‐^ scavenging effect of LDH‐CeO_2_ was also evaluated by ESR spectroscopy.^[^ ^30^ ^]^ As demonstrated by Figure 2d, the characteristic pattern of O_2_^•‐^ are largely weakened by CeO_2_ NPs and LDH‐CeO_2_ under neutral condition. The inhibited O_2_^•‐^ scavenging effect of CeO_2_ NPs in the acidic condition was significantly recovered by the LDH‐CeO_2_ due to the acid neutralization by LDH.^[^ ^16a,b,d^ ^]^ The O_2_^•‐^ inhibition results indicate the similar results as discussed above (Figure 2e). In addition, the inhibition rates of both •OH and O_2_^•‐^ can be significantly augmented by adding LDH‐CeO_2_ into acidic medium (pH 4.5), owing to the anti‐acidification effect by LDH (Figure 2f). The mechanism of such an enhanced ROS‐scavenging capacity by LDH‐CeO_2_ under the acidic condition can be concluded briefly as follows (Figure 2g). i) The catalytic activity of CeO_2_ NPs is much inhibited by excessive H^+^ in acidic condition, which strongly prevents the conversion of Ce^4+^ to Ce^3+^ and the re‐exposure of the active catalytic sites (Figure 2g). Therefore, the redox cycling of CeO_2_ NPs is greatly hindered. ii) The acidity is largely neutralized by LDH after LDH‐CeO_2_ application, thus the redox cycling reactions can be initiated without the suppression by excessive H^+^ (Figure 2g). The acid neutralization effect of LDH strongly facilitates the ROS‐scavenging performance of CeO_2_ NPs in the acidic RA environment, resulting in much enhanced efficacy for treating hyperacidity‐associated disease like RA by LDH‐CeO_2_.

### 2.3 ROS Scavenging and Inflammatory Immune Induction Driven by LDH‐CeO_2_ In Vitro

**Figure 3:**
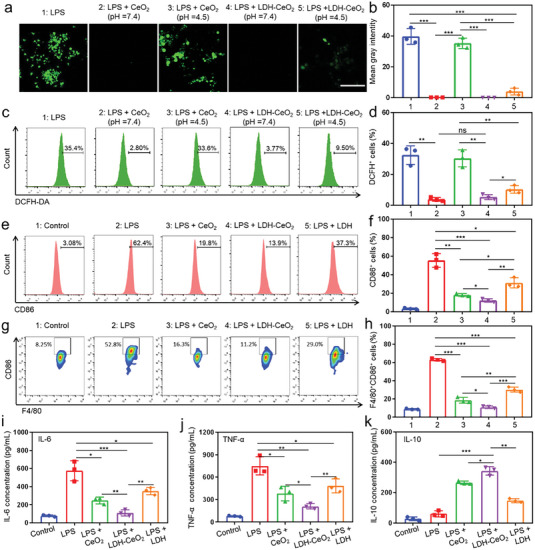
Scavenging of ROS and subsequent anti‐inflammatory immune modulation on macrophages by LDH‐CeO_2_ in vitro. a) Confocal images and b) quantifications of DCFH‐DA^+^ (green) cells incubated with different groups. Scale bar: 50 µm. c) Flow cytometry analyses and d) corresponding quantifications of DCFH‐DA^+^ cells after different treatments. e) Flow cytometry analyses and f) corresponding quantifications of CD86^+^ macrophages treated by various groups. g) Flow cytometry analyses and h) corresponding quantifications of F4/80^+^CD86^+^ BMDMs after different treatments. Cytokines in supernatants collected from different groups measured by ELISA of i) IL‐6, j) TNF‐α and k) IL‐10. All values are presented as means ± s.d, *n* = 3. ^*^ *P* < 0.05, ^**^ *P* < 0.01, ^***^ *P* < 0.001, ns, not significant, two‐tailed Student's *t*‐test.

Macrophages play a central role in the pathogenesis of RA. The pro‐inflammatory M1 macrophages is triggered by systemic or local inflammation, which then further exacerbates the inflammatory microenvironment of RA.^[^ ^4^, ^5^, ^31^ ^]^ Therefore, inducing the anti‐inflammatory phenotype (M2) macrophage polarization is of great importance in suppressing the aggravation of RA. Researches have proved that the high cellular ROS level is largely correlated with the M1 activation of macrophages and the overproduction of ROS is the main cause of synovial inflammation,^[^ ^5^, ^31^ ^]^ thus the ROS scavenging evaluation in vitro is of significance to understand the polarization effect of M2 phenotype macrophage by LDH‐CeO_2_. First, the in vitro biocompatibility of LDH‐CeO_2_ was investigated in L929, standard cell line for investigation of cellular toxicity caused by nanomaterials,^[^ ^32^ ^]^ and RAW264.7 macrophage cell lines. The results indicate that no significant toxicity has been triggered by LDH‐CeO_2_ at the concentrations not higher than 800 ppm in both cell lines (cellular viability is over 80%) (Figure S9, Supporting Information). Besides, the calculated IC50 values are above 1400 ppm even under incubation for as long as 72 h in both cell lines (Table S2, Supporting Information), further verifying the excellent in vitro biocompatibility of LDH‐CeO_2_. Based on the above results, the concentration of LDH‐CeO_2_ used in cellular assay was fixed at 200 ppm, while the pristine LDH is 197 ppm for guaranteeing the same concentration of Mg amount. Then, the internalization of LDH‐CeO_2_ was evaluated in macrophage cell line (RAW 264.7) by confocal laser scanning microscopy (CLSM) and flow cytometry at first. As displayed in Figure S10a (Supporting Information), the fluorescence intensity of LDH‐CeO_2_ (CeO_2_ is labeled by Rhodamine B (RhB)) can be clearly visualized under incubation with RAW 264.7 for 2 and 4 h. The RhB‐positive signals could also be detected in 2 to 4 h of co‐incubation evidenced by flow cytometry, which is consistent to CLSM results (Figure S10b, Supporting Information). The cellular uptake profile indicates that the suitable size distribution and excellent dispersity of LDH‐CeO_2_ are highly favorable for macrophage internalization, facilitating the late depletion of overproduced ROS intracellularly. Considering that the RA microenvironment is always accompanied by hyperacidity (pH ≈5‐6) due to the excessive activation of osteoclasts, we then examined the ROS scavenging efficacy of LDH‐CeO_2_ or CeO_2_ NPs under acidic or neutral condition. As observed under CLSM (**Figure** **3**a), both LDH‐CeO_2_ and CeO_2_ NPs exhibit excellent ROS scavenging efficiency, as confirmed by much‐weakened fluorescence intensity in the 2′,7′‐Dichlorodihydrofluorescein diacetate positive (DCFH‐DA^+^, green) area under neutral condition. In contrast, the CeO_2_ NPs could deplete much less ROS under the acidic condition. Fortunately, LDH‐CeO_2_, even when subjected to acidic condition, performed considerably better in ROS scavenging which is comparable to that under neutral condition (Figure 3a), largely because of the acid neutralization by LDH. The quantifications of CLSM images also give the similar results as above (Figure 3b). In the further investigation by flow cytometry, the DCFH^+^ signal of LDH‐CeO_2_ under acidic condition is much weaker than that of CeO_2_ NPs in acidic condition, which was also comparable to those of LDH‐CeO_2_ and CeO_2_ NPs under neutral condition (Figure 3c). The quantification results reveal the similar behaviors as mentioned above (Figure 3d).

After confirming the ROS scavenging capability, the anti‐inflammatory efficacy of LDH‐CeO_2_ was then examined. As demonstrated in Figure 3e, the CD86‐positive (CD86^+^, representing M1 macrophages) cell percentage is significantly reduced by the LDH‐CeO_2_ compared to that in LPS, CeO_2_ NPs, and LDH groups. As shown by the flow cytometry analysis of anti‐inflammatory effect of Mg ions, the addition of Mg ions also significantly downregulated the expression of CD86 (Figure S11A, Supporting Information), which is further confirmed by the quantification result of CD86^+^ cell percentage (Figure S11B, Supporting Information), suggesting that the significant polarization of macrophages toward M2 phenotype by pristine LDH by the released Mg ions. Therefore, the much‐enhanced induction of M2 polarization by LDH‐CeO_2_ can be attributed to the co‐operative anti‐inflammatory performances by antioxidant CeO_2_ NPs and Mg ions released from LDH.^[^ ^17^ ^]^ The similar conclusion can also be draw from the quantification of CD86^+^ cell percentage (Figure 3f). The flow cytometry analyses further display that LDH‐CeO_2_ and CeO_2_ NPs induce much more significant elevations of CD206‐positive (CD206^+^, representing M2 macrophages) cell amount than that in LPS or LDH group (Figure S12a, Supporting Information), which was also verified by the corresponding quantifications (Figure S12b, Supporting Information). The underlying mechanism for such a M2 polarization of macrophages is the dephosphorylation of nuclear transcriptional factor (NF‐κB), associated with the reduction of intracellular level of ROS by LDH‐CeO_2_ treatment, as evidenced by the significant reduction of phosphorylated NF‐κB (pNF‐κB) detected by western blotting (WB) (Figure S13, Supporting Information).^[^ ^33^ ^]^ Moreover, the molecular pathways of HIF‐1α and pSTAT3 are also associated with ROS, which displayed significant reduction upon LDH‐CeO_2_ treatment (Figure S14, Supporting Information), indicating that multiple macrophage polarization pathways are involved.^[^ ^34^ ^]^ To further investigate the M2 polarization induction effect of LDH‐CeO_2_, the bone marrow derived macrophages (BMDMs) were applied. The flow cytometry results indicate that the LDH‐CeO_2_ induces apparent reduction of F4/80 and CD86 double positive (F4/80^+^CD86^+^, M1 macrophages) cell percentage in comparison to that in LPS, CeO_2_ NPs or LDH groups, which confirms the effective anti‐inflammatory function in the primary cell lines by LDH‐CeO_2_ (Figure 3g,h). Moreover, the most significant enhancement of F4/80 and CD206 double‐positive (F4/80^+^CD206^+^, M2 macrophages) cell percentage was observed in the LDH‐CeO_2_ group, evidencing the effective M2 polarization induced by such nanocatalytic composite (Figure S15a,b, Supporting Information). Besides, the ELISA results show significant expression down‐regulations in inflammatory cytokines of IL‐6 or TNF‐α (Figure 3i,j) and expression upregulation of anti‐inflammatory cytokine of IL‐10 (Figure 3k) by LDH‐CeO_2_, which confirms the validity of modulating the inflammatory immune microenvironment by abundant secretion of anti‐inflammatory cytokines during the re‐programing of macrophages phenotype through efficient ROS scavenging by LDH‐CeO_2_ under acidic RA condition.

### 2.4 Anti‐Inflammatory Modulation by LDH‐CeO_2_ Promotes Osteogenesis while Inhibiting Osteoclastic Activity

**Figure 4:**
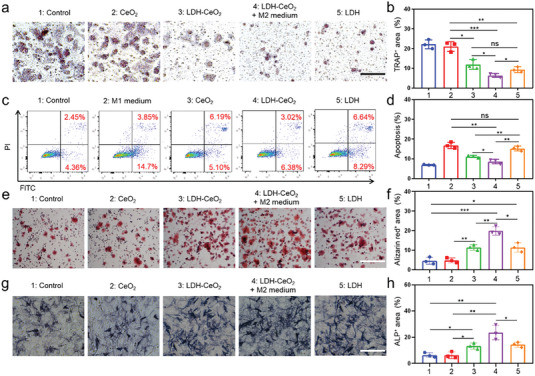
Remodeling the in vitro osteoclastic and osteogenic activity by LDH‐CeO_2_. a) Observations of osteoclast formation (pink) from the fusion of macrophage cells and its prevention by LDH‐CeO_2_ and b) corresponding quantifications. Scale bar: 50 µm. c) Measurements of Osteoblast apoptosis by flow cytometry and d) corresponding quantifications of different treatments. Osteogenesis activity of osteoblasts (MC3T3‐E1) characterized by e) Alizarin red staining and f) corresponding Alizarin red positive area quantifications after various treatments. Scale bar: 100 µm. g) ALP staining and h) ALP positive area quantification after various treatments for analysis of osteogenesis activity of osteoblasts. Scale bar: 100 µm. All values are presented as means ± s.d, n = 3. ^*^ *P* < 0.05, ^**^ *P* < 0.01, ^***^ *P* < 0.001, ns, not significant, two‐tailed Student's *t*‐test.

In above section, we can find that the LDH‐CeO_2_ performs significant induction of M2 macrophages, capable of promoting the bone tissue repair or regeneration by its excellent ROS scavenging capability in both acidic and neutral conditions. Moreover, the Mg ions, a potent agent in osteoclast inhibition and osteogenesis activation, could be released from LDH attributing to the acid neutralization.^[^ ^16^, ^17^, ^18^ ^]^ Therefore, we then evaluated the osteoclastic and osteogenic activities induced by M2 macrophages upon LDH‐CeO_2_ treatment. We discovered that the formation of osteoclasts by the macrophage cell (RAW 264.7) fusion can be much inhibited by LDH‐CeO_2_, and the inhibition effect can be further strengthened by LDH‐CeO_2_ + M2 medium (medium collected from M2 macrophages) (**Figure** **4**a,b), in comparison to that by pristine LDH. Such an enhanced osteoclast inhibition was most probably induced by the co‐effects of Mg ions released from LDH after acid neutralization and anti‐inflammatory cytokines secreted by M2 macrophages.^[^ ^17^, ^28^ ^]^ In addition, considering that the acidic or inflammatory microenvironment is of vital importance for the osteoclast, the viability of osteoclast incubated with LDH‐CeO_2_ or M2 medium was then quantified by flow cytometry through co‐staining with Annexin‐V‐FITC and PI (Figure S16a,b, Supporting Information). Results reveal the apparent apoptosis of osteoclasts by both LDH‐CeO_2_ or LDH‐CeO_2_ + M2 medium, confirming that the bone erosion can be largely prevented by anti‐inflammatory induction as well as inhibition of the osteoclastic activity via acid neutralization.^[^ ^5^, ^16^ ^]^ As investigated by previous researches, the viability of osteoblasts could be remarkably reduced by the M1 macrophages and inflammatory cytokines.^[^ ^4^, ^5^, ^31^ ^]^ Therefore, we then examined whether the LDH‐CeO_2_ could relieve the apoptosis of osteoblast cell line (MC3T3‐E1) or not by flow cytometry. As demonstrated in Figure 4c, the osteoblast apoptosis by M1 macrophages is much mitigated upon LDH‐CeO_2_ addition, suggesting that the LDH‐CeO_2_ could enhance the osteogenesis by preventing the apoptosis of osteoblasts, as also verified by corresponding quantification results (Figure 4d). Besides, LDH‐CeO_2_ is capable of promoting the osteogenesis of MC3T3‐E1, largely attributing to the effect of released Mg ions as confirmed by pristine LDH (Figure 4e),^[^ ^16^, ^17^, ^35^ ^]^ which can be visualized from the biomineralization (calcification) region (red area) stained by alizarin red (Figure 4e) and corresponding quantification results (Figure 4f). The much enhanced osteogenic activities by LDH‐CeO_2_ and LDH‐CeO_2_ + M2 medium were also confirmed by alkaline phosphatase (ALP) staining (Figure 4g), similarly contributed by the released Mg ions (Figure 4g) and the secreted anti‐inflammatory cytokines, as also verified by the quantification result of ALP‐positive (ALP^+^) area (Figure 4h). In summary, LDH‐CeO_2_ is highly effective in reducing the activity of osteoclasts while elevating the viability of osteoblasts and osteogenesis, which may result in much enhanced damaged bone repair.

### 2.5 Therapeutic Efficacy of LDH‐CeO_2_ in Inhibiting RA In Vivo

**Figure 5:**
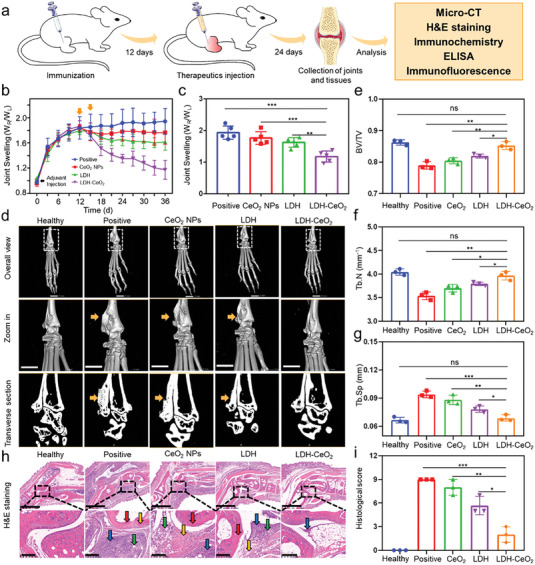
In vivo therapeutic effect of LDH‐CeO_2_ in AIA models. a) Schematic illustration of animal experiment for evaluating the RA therapeutic efficacy of LDH‐CeO_2_. b) Joint swelling measurements of RA model mice during immunization and therapeutic periods. c) Joint swelling quantifications at the end of therapy. d) Representative reconstructed 3D micro‐CT images of joints collected from different groups. Scale bar: 2 mm. Bone related parameters of e) BV/TV, f) Tb. N (mm^−1^) and g) Tb. Sp (mm) calculated from reconstructed images. h) Representative H&E staining sections of joints from different groups. Inflammatory cell infiltration (blue arrow), hyperplastic synovial tissue (orange arrow), bone erosion (red arrow) and invasion of pannus tissues (green arrow). Scale bar: 1.25 mm, 200 µm. i) Calculation result of histological score from H&E sections. All values are presented as means ± s.d, *n* = 3. ^*^ *P* < 0.05, ^**^ *P* < 0.01, ^***^ *P* < 0.001, ns, not significant, two‐tailed Student's *t*‐test.

To verify the therapeutic ability of LDH‐CeO_2_ in vivo, the adjuvant induced arthritis (AIA) animal models were established by the intra‐articular injection of complete Freund's adjuvant at the right hind ankle joints of 10 weeks old female Balb/c mice, followed by 12 days of immunization, and then distinct swelling joints could be observed (**Figure** **5**a). The AIA model was chosen because the joints of the animal suffering AIA share the most pathologic characteristics with human RA, for example, the chronic inflammation and bone or cartilage damages.^[^ ^5^, ^31^ ^]^ AIA mice were separated into 4 groups randomly and the experimental groups were injected with CeO_2_ NPs, LDH and LDH‐CeO_2_ on day 12 and 15. The joint swelling parameters were recorded every 2 days since adjuvant injection. After the end of therapeutic period at day 36, mice were sacrificed and their right hind ankle joint tissues and main organs were harvested for analysis (Figure 5a). Results indicate that CeO_2_ NPs could hardly alleviate joint swelling under the hyperacidity environment of RA (Figure 5b). On the contrary, the administration of LDH‐CeO_2_ resulted in considerable mitigation of joint swelling compared to that in positive control or other therapeutic groups (Figure 5b), which strongly suggest the availability of our microenvironment modulation strategy without generating noticeable fluctuation on body weight of mice (Figure S17, Supporting Information). Interestingly, the pristine LDH group also exhibits moderate alleviation of RA progression compared to positive control (Figure 5b), which is possibly attributed to the acid neutralization by LDH and osteogenic or anti‐inflammatory regulation of released Mg ions in a synergistic manner.^[^ ^16^, ^17^ ^]^ The quantification result on day 36 further verifies the excellent mitigation of joint swelling by LDH‐CeO_2_ (Figure 5c).

Then, the bone related parameters were monitored through micro‐computed tomography (micro‐CT) to analyze the changes in bone microstructure. The micro‐CT images of joint reveal the severe bone erosion or damage (rough surface or abnormal trabecular structure indicated by orange arrow) in positive control, while the pristine CeO_2_ NPs injection fail to ameliorate the symptoms related to bone damage. In contrast, the administration of LDH‐CeO_2_ resulted in the desirable therapeutic outcome and no significant or negligible bone abnormal (smooth bone surface or normal trabecular structure) could be visualized, which is almost comparable to the healthy mouse's joint (Figure 5d). The micro‐CT result also indicates that the LDH is capable of alleviating the bone damage moderately, possibly contributed by the acid neutralization and Mg ion released from the LDH (Figure 5d). Quantification analysis of bone‐related parameters from micro‐CT presents that the amount of bone per tissue volume (BV/TV) (Figure 5e), trabecular number (Tb. N mm^−1^) (Figure 5f) and trabecular separation (Tb. Sp mm) (Figure 5g) have been improved markedly by LDH‐CeO_2_ without noticeable differences with healthy ones. It is also worth noticing that LDH‐CeO_2_ treatment has also notably elevated bone mineral density (BMD, mgHA/cm^3^) (Figure S18a, Supporting Information) and trabecular thickness (Tb. Th, mm) (Figure S18b, Supporting Information) values of joints from treated mice to the levels analogous to those in healthy mice.

For more specific evaluation on therapeutic efficacy of LDH‐CeO_2_, the histological analysis was then conducted. The H&E sections (Figure 5h) show the massive inflammatory cell infiltration (blue arrow), serious hyperplastic synovial tissue (orange arrow), bone erosion (red arrow) and invasion of pannus tissues (green arrow) in joint cavity in both positive control and CeO_2_ group.^[^ ^31^, ^36^ ^]^ However, those abnormalities have been notably weakened by LDH‐CeO_2_ treatment, and only slight inflammatory cell infiltration and the intact bone morphology in comparison to that in healthy control can be observed (Figure 5h), verifying the superior anti‐inflammatory regulation effects of our prepared nanocatalytic platform. Besides, the H&E sections from LDH‐treated mice also show moderate alleviation of RA pathogenesis, revealing the significant anti‐inflammatory capability owing to the Mg ion release and hyperacidity neutralization by LDH (Figure 5h). The detailed histological score also evidences the considerable therapeutic efficacy of LDH‐CeO_2_ as depicted above (Figure 5i).^[^ ^36^ ^]^ Proteoglycan loss and cartilage erosion are always generated by RA. Therefore, to evaluate the therapeutic effect of LDH‐CeO_2_ on RA regrading to cartilage damage, safranin O and fast green (SO‐FG) staining were conducted. The SO‐FG staining sections from positive and CeO_2_ groups show serious proteoglycan loss and cartilage erosion, judged from the hardly observable red color (representing the cartilage) and smooth surface (Figure S19a, Supporting Information). Thankfully, the injection of LDH‐CeO_2_ induced substantial reversal of cartilage damage caused by RA, which is revealed by the appearance of large proportion of red color area and smooth surface, indicating the protection and/or regeneration of cartilage after the treatments by LDH‐CeO_2_ (Figure S19a, Supporting Information).^[^ ^5^, ^37^ ^]^ The quantifications of SO positive areas (Figure S19b, Supporting Information) demonstrate the similar results as presented above.

### 2.6 LDH‐CeO_2_ Reshapes the Inflammatory Immune Microenvironment of RA

**Figure 6:**
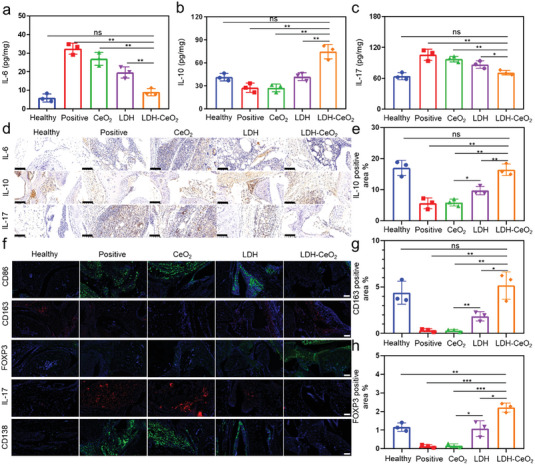
Reshaping the RA immune microenvironment by the immunomodulatory effect of LDH‐CeO_2_. Cytokine levels analyzed by ELISA of a) IL‐6, b) IL‐10, and c) IL‐17 in the joint tissues collected from treated mice. d) Immunochemical staining images of cytokine (IL‐6, IL‐10, and IL‐17) expressions in joints. Scale bar: 100 µm. e) Quantifications of IL‐10 positive area of joint sections. f) Immunofluorescence staining (CD86: green, CD163: red, FOXP3: green, IL‐17: red, CD138: green) images of the joints for monitoring activities of immune cells after different treatments. Scale bar: 50 µm. g) CD163 and h) FOXP3 positive area quantification of immunofluorescence staining images. All values are presented as means ± s.d, *n* = 3. ^*^ *P* < 0.05, ^**^ *P* < 0.01, ^***^ *P* < 0.001, ns, not significant, two‐tailed Student's *t*‐test.

As proved in vitro that the LDH‐CeO_2_ performed excellent M2 polarization induction in macrophages, we further investigated that whether the excellent RA inhibition resulted from macrophage regulation and the sequential immune modulation or not. The ELISA results demonstrate that the expression of IL‐6 cytokines is significantly down‐regulated, evidencing the marked inhibition of M1 macrophage polarization by LDH‐CeO_2_ (**Figure** **6**a).^[^ ^38^ ^]^ While, the IL‐10 cytokine expression is up‐regulated notably by LDH‐CeO_2_ treatment, attributable to the much‐enhanced proportion of M2 macrophages and the recruitment of Treg cells (Figure 6b), thus resulting in the considerable suppression of Th 17 cells (marked decreasing of IL‐17 cytokine) (Figure 6c) and plasma cells (notable reduction of IgM antibody) (Figure S20a, Supporting Information).^[^ ^4^, ^39^ ^]^ The significant changes in cytokine or autoimmune antibody expressions were also confirmed by the immunochemical staining shown in Figure 6d and Figure S20b (Supporting Information). In addition, the quantification of IL‐10 positive area in the immunochemical staining section displays a notable up‐regulation, again verifying the significant anti‐inflammatory regulation of LDH‐CeO_2_ (Figure 6e). Moreover, the quantifications of other cytokine (IL‐6 or IL‐17) positive areas show similar trends as above (Figure S21, Supporting Information). These results suggest that the inflammatory microenvironment of RA could be efficiently reshaped by anti‐inflammatory cytokine release, Treg cell recruitment and Th 17 and/or plasma cell inhibitions by polarizing the macrophages in RA joints into M2 phenotype.

For more evident surveillance of immune cell alternation in joints, the immunofluorescence staining was conducted. The result indicates that the proportion of M1 macrophages (CD86^+^, green) was dramatically reduced, accompanied by the significant proportion elevation of M2 macrophages (CD163^+^, red) after LDH‐CeO_2_ administration (Figure 6f), as further confirmed by the quantification of fluorescence intensity (Figure S22a, Supporting Information; Figure 6g), revealing the desirable M2 macrophages induction capacity of such a nanocatalytic platform. The Treg cell cohort (FOXP3^+^, green), however, has also performed a significant elevation upon LDH‐CeO_2_ treatment (Figure 6f,h), indicating that the LDH‐CeO_2_ is capable of modulating the inflammatory microenvironment of RA by activating M2 macrophages (Figure 6f,g) and recruiting Treg cells, therefore resulting in the significant suppression of Th 17 cells (IL‐17^+^, red, Figure 6f) (Figure S22b, Supporting Information). Furthermore, the anti‐inflammatory remodulation by LDH‐CeO_2_ down‐regulates the activity of plasma cells (CD138^+^, green) (Figure 6f; Figure S22c, Supporting Information), resulting in the much lowered autoimmune antibody production as displayed in Figure S20 (Supporting Information), which further evidences favorable inflammatory immune inhibition and damaged bone healing effects of the treatment. In conclusion, the LDH‐CeO_2_ not only exhibits excellent M2 macrophage induction effect by ROS scavenging via acidity‐neutralizing, but also plays an in situ immune regulatory role in the treatment, thus reshaping the inflammatory microenvironment in RA mice model, and promoting the healing of damaged joints.

To evaluate the in vivo safty, the actue toxicity of LDH‐CeO_2_ was assessed for obtaining the LD50 value.^[^ ^40^ ^]^ As listed in Table S2 (Supporting Information), five groups of mice were injected intravenously in five varied doses. Then, the plot of the probit value against the log dose was fitted and used to calculate the Log dose corresponding to probit 5 (50% of death) (Figure S23, Supporting Information). Thus, the Log LD50 has been calculated to be 2.667 and the corresponding LD50 value of LDH‐CeO_2_ is ≈480 mg k^−1^g. This LD50 is much higher than the therapeutic dosage (30 mg k^−1^ g), indicating the excellent biocompatibility and safety of this RA therapeutic modality by LDH‐CeO_2_. Additionally, H&E staining indicates no distinct pathological changes in major organs (hearts, livers, spleens, lungs, and kidneys) from LDH‐CeO_2_ treated mice (Figure S24, Supporting Information), while the hematological liver functional parameters (alanine aminotransferase (ALT), aspartate aminotransferase (AST), alkaline phosphatase (ALP)) (Figure S25a–c, Supporting Information) as well as kidney functional parameters (blood urea nitrogen (BUN) and creatinine (Cr)) of experimental mice, all list among the normal expression levels (dotted lines) (Figure S25d,e, Supporting Information), demonstrating the favorable compatibility and negligible side effect of LDH‐CeO_2_ during the RA treatment. Furthermore, all the blood routine results from LDH‐CeO_2_ groups are not significantly different from those in other groups (Figure S26, Supporting Information), which confirms the favorable systemic safety of our formulation.

## 3 Conclusion

To overcome the current limitation in traditional RA therapy, a novel therapeutic strategy for RA based on nanocatalytic material LDH‐CeO_2_ has been proposed. The constructed inorganic nano‐platform triggers notable M2 polarization of macrophages by the enhanced ROS‐scavenging activity of CeO_2_ NPs through the acid neutralization by LDH at the RA site, thus further remodulating the inflammatory microenvironment by recruiting the immune suppression Treg cells while inhibiting the Th 17 and plasma cells. Meanwhile, the Mg ion released from degraded LDH suppresses the formation of osteoclasts by inhibiting monocyte fusion, therefore largely inhibit preventing the bone erosion. More importantly, the viability and osteogenesis of osteoblasts are significantly elevated by the inhibited expressions of inflammatory cytokines and autoimmune antibody, and of the released Mg ion, resulting in the profound healing of bone tissues. In summary, the present nanocatalytic platform exhibits highly encouraging therapeutic outcome for RA by the in situ induced modulation of immune microenvironment and bone healing in a synergistic manner, thus presenting a promising prospect for treating RA clinically.

## Conflict of Interest

The authors declare no conflict of interest.

## Supporting information

Supporting Information

## Data Availability

The data that support the findings of this study are available from the corresponding author upon reasonable request.
