# Supplementary material for: Anti‐Acidification and Immune Regulation by Nano‐Ceria‐Loaded Mg–Al Layered Double Hydroxide for Rheumatoid Arthritis Therapy
Source: Adv Sci (Weinh). 2023 Dec 8;11(6):2307094. doi: 10.1002/advs.202307094 (PMC10853726; doi:10.1002/advs.202307094)
Supplement: Supplementary file 1 — Supporting Information [file ADVS-11-2307094-s001.pdf]

ADVANCED SCIENCE
Open Access

Supporting Information

for *Adv. Sci.*, DOI 10.1002/advs.202307094

Anti-Acidification and Immune Regulation by Nano-Ceria-Loaded Mg–Al Layered Double Hydroxide for Rheumatoid Arthritis Therapy

Hao Fu, Yuedong Guo, Wenming Fang, Jiaxing Wang, Ping Hu* and Jianlin Shi**

Supporting information

Anti-acidification and Immune Regulation by Nano-Ceria-Loaded Magnesium-Aluminum Layered Double Hydroxide for Rheumatoid Arthritis Therapy

Hao Fu, Yuedong Guo, Wenming Fang, Jiaxing Wang*, Ping Hu*, Jianlin Shi*

Dr. H. Fu, Dr. W. Fang
Shanghai Institute of Ceramics, Chinese Academy of Sciences, Research Unit of Nanocatalytic Medicine in Specific Therapy for Serious Disease, Chinese Academy of Medical Sciences (2021RU012), Shanghai 200050, P. R. China.

Dr. Y. Guo
Platform of Nanomedicine Translation, Shanghai Tenth People's Hospital, Medical School of Tongji University, 38 Yun-xin Road, Shanghai 200435, P.R. China.

Prof. J. Wang
Department of Orthopaedics, Shanghai Jiao Tong University Affiliated Sixth People’s Hospital, Shanghai Jiao Tong University, Shanghai 200233, P.R. China
E-mail: jxwang@shsmu.edu.cn

Prof. P. Hu, Prof. J. Shi
Shanghai Institute of Ceramics, Chinese Academy of Sciences, Research Unit of Nanocatalytic Medicine in Specific Therapy for Serious Disease, Chinese Academy of Medical Sciences (2021RU012), Shanghai 200050, P. R. China.
Platform of Nanomedicine Translation, Shanghai Tenth People's Hospital, Medical School of Tongji University, 38 Yun-xin Road, Shanghai 200435, P.R. China.
E-mail: huping@mail.sic.ac.cn, jlshi@mail.sic.ac.cn

Experimental Section

Materials, chemicals and reagents

Magnesium nitrate hexahydrate ($Mg(NO_3)_2 \cdot 6H_2O$), aluminum nitrate nonahydrate ($Al(NO_3)_3 \cdot 9H_2O$) were obtained from Aladdin (China). Cerium(III) nitrate hexahydrate ($Ce(NO_3)_3$) was purchased from Sigma-Aldarich (USA). $NaHCO_3$, NaOH, HCl were obtained from Sinopharm Chemical Reagent (China). Trichloromethane (99.0%), toluene (99.5%) tert-butylamine (99.5%) and anhydrous ethanol (99.7%) and methyl blue (MB) were purchased from Shanghai Lingfeng Chemical Reagent (China). DSPE-PEG_{2k}-COOH was bought from the Ponsure (China). Cellular ROS kit (DCFH-DA), CCK-8, DAPI, Annexin-5 FITC/PI were purchased from Beyotime Biotechnology (China). Alizarin Red S solution, SOD assay kit and catalase assay kit were purchased from Solarbio (China). Fetal bovine serum (FBS), 0.25% trypsin-EDTA solution and 1% penicillin/streptomycin solution were purchased from Gibco (USA). Dulbecco's modified eagle's medium (DMEM, high glucose), alpha-modified Eagle's medium (α-MEM) and phosphate buffered saline solution (PBS, pH = 7.4, 6.5 and 4.5) were acquired from YOBIBIO. Oleic acid (90%) and ALP staining kit was purchased form Wako-chemical (Japan). Lipopolysaccharide (LPS) was obtained from MCE (USA). The cytokines RANKL and M-CSF were provided by R&D Systems (USA). Cytokines (IL-6, TNF-α, IL-10, IL-17) and autoimmune antibody (IgM) ELISA kits were acquired from Mlbio (China). Flow cytometry antibodies CD86 (FITC) were obtained from BD biosciences, while CD206 (APC anti-mouse CD206, clone C068C2), F4/80 (PE anti-mouse F4/80, clone BM8) were purchased from BioLegend. The immunochemical staining and immunofluorescence antibody CD86 (19589) and FOXP3 (72338) was purchased from CST (USA); IL-17 (bs-1183r) and IgM (bs-0368R) were bought from Bioss (China), while the CD163 (ab182422), IL-10 (ab217941), IL-6 ((ab290735)) and CD138 (ab128936) were obtained from Abcam (USA).

Preparation of CeO_2-OA and CeO_2-PEG

The $Ce(NO_3)_3$ (0.107g) was dissolved in 15 mL deionized H_2O and then added into 50 mL hydrothermal kettle. After that, the mixture of toluene (14.25 mL), oleic acid (0.6 mL) and tert-butylamine (0.15 mL) was added into the autoclave in open air without stirring. Then, the sealed autoclave was heated in the oven at 180°C for 36 h. The obtained product is CeO_2-OA. For improving the dispersity in aqueous phase and biocompatibility for biomedical application, CeO_2-OA was further modified by DSPE-PEG_{2k}-COOH. Briefly, 2 mL of trichloromethane containing 8 mg of CeO_2-OA were mixed with 10 mL of trichloromethane containing 40 mg of DSPE-PEG_{2k}-COOH and the mixture was subjected by ultrasonic shaking. After mixture of trichloromethane was completely vaporized by rotary evaporation under 60°C water bath, deionized H_2O was added accompanying by ultrasonic mixing and then the CeO_2-OA modified by DSPE-PEG_{2k}-COOH (CeO_2-PEG) was obtained.

Preparation of MgAl-LDH

$Mg(NO_3)_2 \cdot 6H_2O$ (3.0 mmol) and $Al(NO_3)_3 \cdot 9H_2O$ (1.0 mmol) was first dissolved in 10 mL deionized H_2O and then quickly dropped into the NaOH solution (0.8g NaOH dissolved in 40 mL deionized H_2O) and placed in magnetic stirring for 30 min at room temperature. After that, the mixed solution containing MgAl-LDH precursors was washed with deionized water for 3 times after centrifugation (11000 rpm, 10 min). Then, the MgAl-LDH precursors solution was again dispersed in 40 mL of deionized H_2O and heated to 100°C for 12 h in a Teflon lining sealed in hydrothermal kettle. The MgAl-LDH were obtained after centrifugation (11000 rpm, 10 min) and washed by deionized H_2O for 3 times.

Preparation of LDH-CeO_2 as a nanocatalytic medicine

The CeO_2-PEG NPs (1 mg/mL) was gradually added into the as-prepared MgAl-LDH suspensions (concentration of 1 mg/mL) followed by the continuous magnetic stirring for 24 h. The resulting LDH-CeO_2 solution was centrifuged at 11000 rpm for 10 min and washed with deionized water 3 times. The obtained LDH-CeO_2 were resuspended

in deionized water for further characterization and experiments.

Characterization

Transmission electron microscopy (TEM) images and selected area electron diffraction (SAED) patterns were obtained by Magellan 400 electron microscope (FEI, USA). Cs-corrected Transmission electron microscopy images and element mappings were acquired using a JEM-ARM300F electron microscope (JEOL, Japan). The XRD patterns were acquired on a Rigaku D/MAX-2250 V diffractometer (40 kV, 40 mA) with a Cu Ka radiation target. Zeta potential was performed using zeta-nanosizer ZS90 (Malvern PANalytical, UK). XPS spectra were recorded by ESCAlab250 (Thermal Scientific, USA). FTIR spectra were measured on Nicolet iS 10 (Thermo Scientific, USA). The concentrations of Mg and Ce ions in PBS solution with different pH values were measured by inductively coupled plasma optical emission spectrometer (ICP-OES). The pH value in solutions were tested by pH meter (PHB-1, SANXIN, China). UV-vis absorption spectra of MB were recorded by a UV-3101PC spectrometer (Shimadzu, Japan). Electron spin resonance (ESR) spectroscopy was recorded on EMXplus spectrometer (Thermo Scientific, USA).

Investigation of antioxidant performance of LDH-CeO_2 and related mechanism

To evaluate the hydroxyl radicals (•OH) production, the electron spin resonance (ESR) spectroscopy was used with the assistance of 5,5-dimethyl-1-pyrroline-N-oxide (DMPO), a typical capture agent for short-lived •OH. The 2 mM H_2O_2, 10 μM $FeSO_4$, 1.5 mM DMPO and LDH-CeO_2 or CeO_2 were added into the PBS with various pH (pH = 4.5, 6.5 or 7.4) values. Then the mixed solution (100 μL) was subjected to ESR measurements. UV-vis absorption spectra, indicating decolorization of methyl blue (MB) caused by •OH catalyzed by ZCMFP, were recorded by a UV-3101PC spectrometer (Shimadzu, Japan). For evaluating the $O_2^{\bullet -}$ scavenging assay, 1 mM xanthine, 0.2 unit/mL XO, 5 mM DMPO and LDH-CeO_2 or CeO_2 were added into the PBS with various pH (pH = 4.5, 6.5 or 7.4) values. Subsequently, the mixed solution

(100 μL) was transferred into the quartz tube for ESR measurements. While the CAT and SOD assay kit at acidic or neutral condition (pH = 4.5, 6.5 or 7.4) according to the manufacturer's protocols.

Acid neutralization capability of LDH-CeO_2

Briefly, PBS solutions (pH 4.5 or 6.5) were titrated with LDH-CeO_2 (50 mg/mL), and NaOH (0.01 or 0.1M) solutions (10 μL per titration) with stirring. The pH values were continuously monitored by a pH meter after each titration (PHB-1, SANXIN, China). The titration was stopped when the titrated solutions experienced more than three consecutive pH decreases of less than 0.01. Moreover, LDH-CeO_2 (50 mg/mL), PBS (pH 7.4), deionized water, CeO_2-PEG (10 mg/mL) and $NaHCO_3$ (1 M) solutions as comparations, were titrated with 1% hydrochloric acid (10 μL per titration) with stirring. The procedures were similar to that mentioned above.

Cell culture

Mouse osteoblast cell line (MC3T3-E1) and mouse fibroblast cell line (L929) were obtained from the cell bank of the Chinese Academy of Sciences (Shanghai). Mouse macrophage cell line (RAW 264.7) was purchased from Procell (China). MC3T3-E1 was cultured in alpha-modified Eagle's medium (α-MEM, Gibco, USA) containing 10% fetal bovine serum (FBS, Gibco, USA) and 1% penicillin and streptomycin (Gibco, USA). The L929 cells were cultured in the DMEM containing 10% fetal bovine serum (FBS, Gibco, USA), while the RAW 264.7 was cultured in the complete macrophage culture medium obtained from Procell (China). All cells were cultured under the condition of 5% CO_2 at 37°C.

In vitro cellular uptake of LDH-CeO_2

5 mg of Rhodamine B (RhB), 8 mg of CeO_2-OA were dissolved with 2 mL of trichloromethane and mixed with 10 mL of trichloromethane containing 40 mg of DSPE-PEG_{2k}-COOH and further was subjected by ultrasonic mixing. After that, the

trichloromethane was completely vaporized by rotary evaporation under 60°C water bath. Then, deionized H_2O was added, accompanied by ultrasonic mixing, and the CeO_2-PEG modified by RhB (CeO_2-PEG-RhB) was obtained. After being washed by PBS 3 times, CeO_2-PEG-RhB were dispersed in DMEM or 1640 culture medium. To examine cellular uptake, RAW 264.7 cells were seeded into 6-well microplates with a density of 10^5/well and cultured overnight under 37°C and 5% CO_2. Then the culture medium was changed with as-prepared 1640 containing CeO_2-PEG-RhB. After co-incubation for 1, 2, and 4 h, RAW 264.7 cells were washed with PBS for 3 times and observed under a confocal laser scanning microscope (CLSM) (Olympus FV1000, Japan). After observation, cells were collected and analyzed by flow cytometry (LSRFortessa, Biosciences, USA).

In vitro biocompatibility evaluation of LDH-CeO_2

To study the influence on cellular viability caused by LDH-CeO_2, the CCK-8 assay was conducted. Briefly, 4×10^3 cells (L929 or RAW264.7) were added in 96-well plates and grown overnight. After the addition of LDH-CeO_2 in different concentrations, cells were incubated for 24, 48, and 72h. After that, 10 μL of CCK-8 solution (Yeasen, Shanghai, China) was added to each well and the plates were incubated at 37°C for 1 h. Finally, the absorbance was measured at 450 nm using a microplate reader. The IC50 values were calculated by SPSS software.

Examination of ROS scavenging efficacy in macrophages

The ROS was determined by fluorescence microscopy and flow cytometry. The macrophages RAW 264.7 were seeded in a 6-well culture plate at a density of 2×10^5 cells/well overnight. After incubated with LPS for 24 h, macrophages were treated with different agents under various pH values for another 24 h. Then, cell culture medium was removed and incubated with DCFH-DA solution (5 mg/mL) for at 37°C for 30 min. After being washed for 3 times with PBS, the culture dishes were observed with a confocal fluorescence microscope (Ex/Em of 488/525 nm) (Olympus FV1000, Japan).

The ROS level was also quantified by flow cytometry. After incubating for 30 min, the cell dishes were washed for 3 times and then detached by trypsin and analyzed by flow cytometry (BD fortessa, USA).

Investigation of M2 repolarization induction efficacy of LDH-CeO_2 in vitro

RAW 264.7 cells were seeded in 6 well plates with density of 2×10^5 cells/well and cultured under 37°C and 5% CO_2. After incubating with LPS (100 ng/mL) for 24 h, LDH-CeO_2 or CeO_2 was added and incubated for another 24 h. After incubation, the RAW 264.7 cells were collected and then subjected to flow cytometry analysis after being stained with antibodies (FITC-CD86, APC-CD206). The $CD86^+$ or $CD206^+$ cell percents were calculated by Flowjo software. For quantifying the anti-inflammatory ability of Mg ions, seeded RAW 264.7 cells were treated with LPS and Mg ion (15 ppm) for 24 h. Then, cells were collected and subjected to flow cytometry analysis after being stained with FITC-CD86. The $CD86^+$ or $CD206^+$ cell percents were calculated by Flowjo software. To further verify the M2 induction ability of LDH-CeO_2, bone marrow-derived mononuclear macrophages (BMDMs) were applied. For the culture of BMDMs, healthy Balb/c mice (4 weeks old) were sacrificed by cervical dislocation. Then, the femur and tibia were taken, followed by the syringing of the bone marrow. The mononuclear cells were separated after lysis of red blood cells. The separated mononuclear cells were then divided into 3 wells in a 6-well culture plate and cultured for 7 days. The complete medium was added to each well to 4 mL, and the final concentration of M-CSF was 10 ng/mL. After incubation with LDH-CeO_2 or CeO_2, BMDMs were washed and collected for staining of antibodies (PE-F4/80, FITC-CD86, APC-CD206). After being subjected to flow cytometry analysis, the $F4/80^+CD86^+$ or $F4/80^+CD206^+$ cell percent were obtained by Flowjo software.

Measurement of immune-related cytokines

The cell supernatants were collected from cells treated by different agents. Then, the cellular levels of IL-6, IL-17, and IL-10 were measured with ELISA kits (Mlbio, China)

according to the manufacturer's instructions.

Measurement of osteoclast activity and viability

For induction of osteoclasts, RAW 264.7 cells were seeded in a 12-well plate at a density of 1×10^4/well. After 24 h, the medium was changed to DMEM medium containing 50 ng/mL RANKL (R&D Systems, USA) and 25 ng/mL M-CSF (R&D Systems, USA). The medium was changed every 2 or 3 days, and TRAP staining (Wako-chemical, Japan) was performed after 7 days of culture. The medium was changed every 2 or 3 days, and TRAP staining (Wako-chemical, Japan) was performed after 7 days of culture. After washing 3 times with PBS (Gibco, USA), it was fixed with 4% paraformaldehyde (PFA) for 30 min, and the operation was performed according to the manufacturer's instruction of TRAP staining kit. Images were taken by microscope and quantification of TRAP-positive area was conducted through Image J software (version 1.53e)

For apoptosis analysis, the osteoclasts were treated with different conditions for 48 h including PBS, CeO_2, LDH-CeO_2, LDH-CeO_2 + M2 medium and pristine LDH respectively. Collected cells and cell supernatants were then put into 500 μL of staining buffer with 5 μL Annexin-5 FITC and 5 μL PI solution. After 30 min of staining at room temperature, cell apoptosis was examined using a flow cytometer (BD LSRFortessa, BD Biosciences, USA).

Evaluations of osteogenic activity of osteoblasts

MC3T3-E1 cells were seeded in a 12-well plate (5×10^4/well), and the cell culture medium was replaced with osteogenic ingredients (50 ng/mL ascorbic acid, 10 mM sodium β-glycerophosphate, 10 nM dexamethasone) culture medium, with DMEM as a control group, and the medium was changed every two to three days. After 7 days of culture under different treatments, an ALP staining kit (Wako-chemical, Japan) was used to detect osteogenic activity according to instruction of manufacturer. Images were observed and taken under an inverted microscope. For quantitative analysis of the ALP

positive area, images were taken by microscope, and the ALP positive area were courted through Image J software (version 1.53e).

MC3T3-E1 cells were seeded in 12-well plate with a seeding density of 5×10^4/well. After the cells were adhered to the wall, the cell culture medium was replaced by osteogenic ingredients every three days. After 7 days of culture, the culture was terminated, and the culture plate was taken out and stained with Alizarin Red. Images were taken by microscope and quantification of the Alizarin Red positive area was conducted through Image J software (version 1.53e).

Evaluation of RA inhibition effectiveness of LDH-CeO_2

The RA models were established by injecting the complete Freud's adjuvant intra-articularlly into right hind ankle joints of 10 weeks old female Balb/c mice, following by 12 days of immunization until distinct swelling joints could be observed. Mice bearing RA were separated into 4 groups randomly. The experimental groups were injected with CeO_2 NPs (0.4 mg/kg), LDH (29.6 mg/kg) and LDH-CeO_2 (30 mg/kg) at day 12 and 15. The joint swelling parameters were recorded every 2 days since adjuvant injection. After the whole therapeutic period of 36 days, mice were sacrificed, and their right hind ankle joint tissues, as well as main organs and peripheral blood, were harvested. The bone morphology of joints was reconstructed by Micro Computer Tomography (micro-CT, VENUS 001, PINGSENG Healthcare, China) and related bone health parameters were obtained therein by reconstruction and analysis software (Avatar3). After micro-CT analysis, joint samples were subjected to decalcification process. After rinsing the decalcification solution, the decalcified bone tissues were dehydrated in gradient ethanol and embedded in wax and then sliced on a Leica microtome with a thickness of 5 μm. Then, the joints sections were subjected to H&E staining. All animal procedures were performed under the guidelines approved by the Institutional Animal Care and Use Committee of the Shanghai Tenth People's Hospital, Medical School of Tongji University (approval no. SHDSYY-2023-Z0026-3).

Evaluation of changes in immune cell cohorts modulated by LDH-CeO_2

To investigate the modulation efficacy of immune cells by LDH-CeO_2, the ELISA analysis of cytokines (IL-6, IL-17, IL-10) or autoimmune antibody (IgM) level in joint tissues was first performed according to manufacturers' protocol. Then, the immunohistochemical staining was conducted by applying IL-6, IL-17, IL-10 and IgM antibodies for quantifying the variations of cytokines or autoimmune antibody in situ. The immunochemical staining sections were scanned by the digital pathology slide scanner (KFBIO, KF-PRO-120). For orthotopic surveillance of immune cell changes, the immunofluorescence staining was further conducted. The joint sections were stained by CD86 (M1 macrophages marker), CD163 (M2 macrophages marker), Foxp3 (Treg cell marker), IL-17 (Th 17 cell marker) and CD138 (plasma cell marker) antibodies for distinguishing the phenotype of immune cells. The stained sections were scanned by the TissueFAXS Plus (version 7.1, TissueGnostics GmbH, Vienna, Austria). For quantitative analysis of cytokines/autoimmune antibodies or certain immune cells positive area, images were scanned and corresponding positive area were courted through Image J software (version 1.53e).

Biocompatibility evaluation and systemic toxicity of LDH-CeO_2

At the end of the therapy period described previously, RA model mice were sacrificed, main organs (heart, liver, spleen, lung, kidney), peripheral whole blood and blood serum were collected and separated. Then, the hematological parameters (alanine aminotransferase (ALT), aspartate aminotransferase (AST), alkaline phosphatase (ALP), blood urea nitrogen (BUN) and creatinine (Cr)) were analyzed according to the whole blood and blood serum samples by automatic biochemistry analyzer (Chemray 240/420/800, Rayto, China). While the examination of blood routine is conducted by the Automatic Blood Cell Analyzer (MindRay, BC-2800vet) by using the collected whole blood treated with an anti-coagulant. The histological changes were observed and analyzed based on H&E staining of main organs. The LD50 value was obtained by following the "staircase method" proposed by Miller and Tainter et al., via using a small

number of animals (2 per dose) and increasing the dose of the drug step-by-step. Then, from no death to 100% death, five doses are selected for determining LD50. During our estimation, five groups of mice were injected intravenously in five varied doses. Animals were observed for any poisoning symptoms in the first 2 hours and then in 6 and 24 hours. In 24 hours, the death rate of mice in each group was calculated. Plot the probit value against the log dose and find the dose corresponding to probit 5, i.e., 50% of death.

Statistical Analysis

All quantitative data are presented as the mean ± standard deviation. The statistical significance of all experiments was determined by Student t test. P-values were considered statistically significant: $^*P < 0.05$, $^{**}P < 0.01$, $^{***}P < 0.001$.

Supplementary Figures

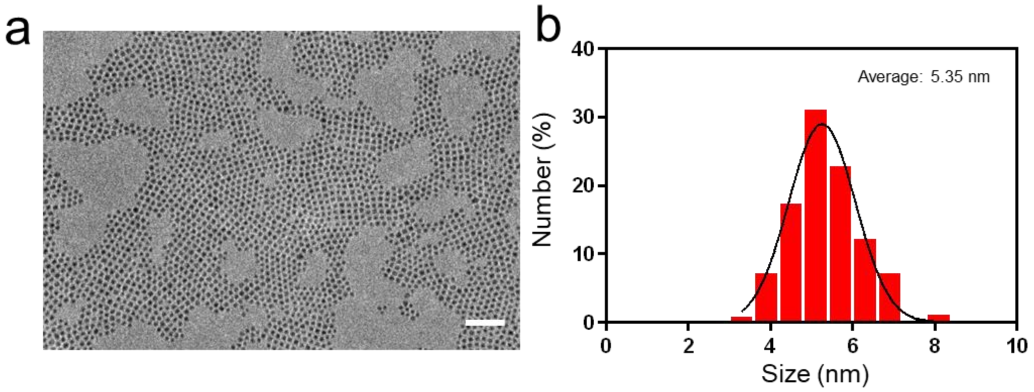

Figure S1. (a) TEM image of CeO_2-OA and (b) corresponding size distribution. Scale bar: 100 nm.

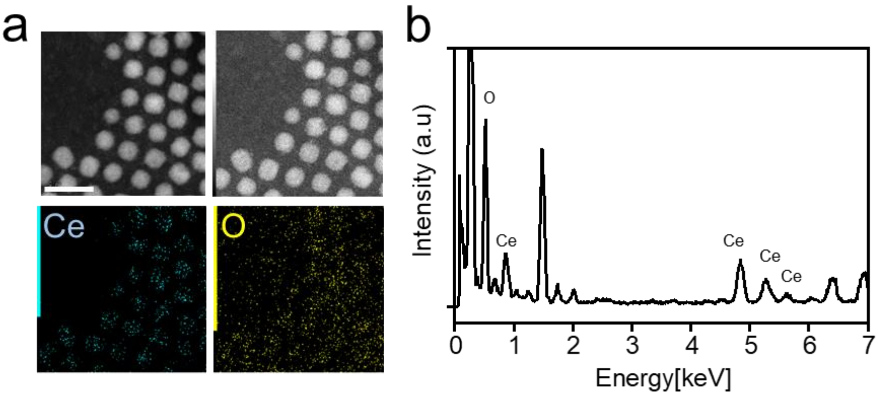

Figure S2. (a) Elemental mapping of CeO_2-OA and (b) corresponding EDS spectra. Scale bar: 5 nm.

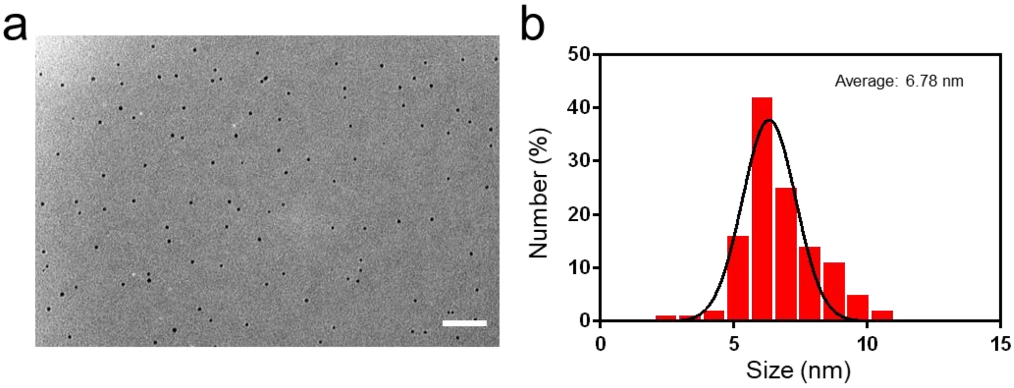

Figure S3. (a) TEM image of CeO_2-PEG and (b) corresponding size distribution. Scale bar: 100 nm.

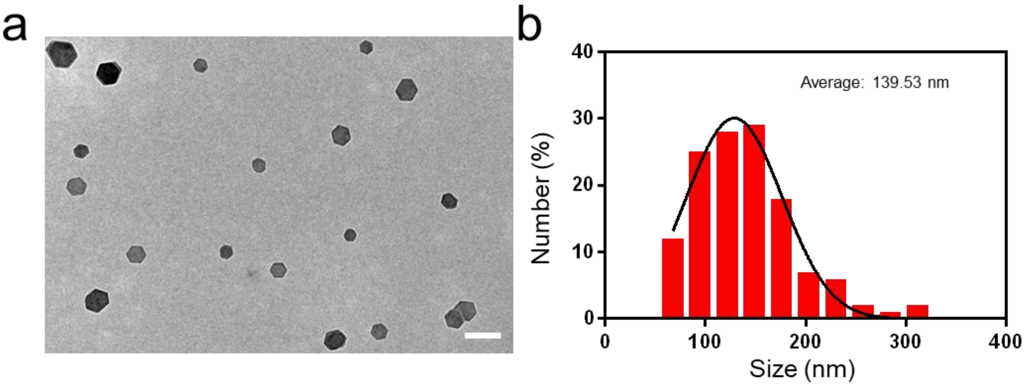

Figure S4. (a) TEM image of MgAl-LDH and (b) corresponding size distribution. Scale bar: 200 nm.

Element	Wt% (ICP-OES)	n (Mg/Al/Ce)
Mg	7.31 ± 0.05	26 : 10 : 1
Al	2.93 ± 0.02	
Ce	1.42 ± 0.04	

Table S1. Analysis of Mg, Al and Ce concentrations in LDH-CeO_2 by ICP-OES.

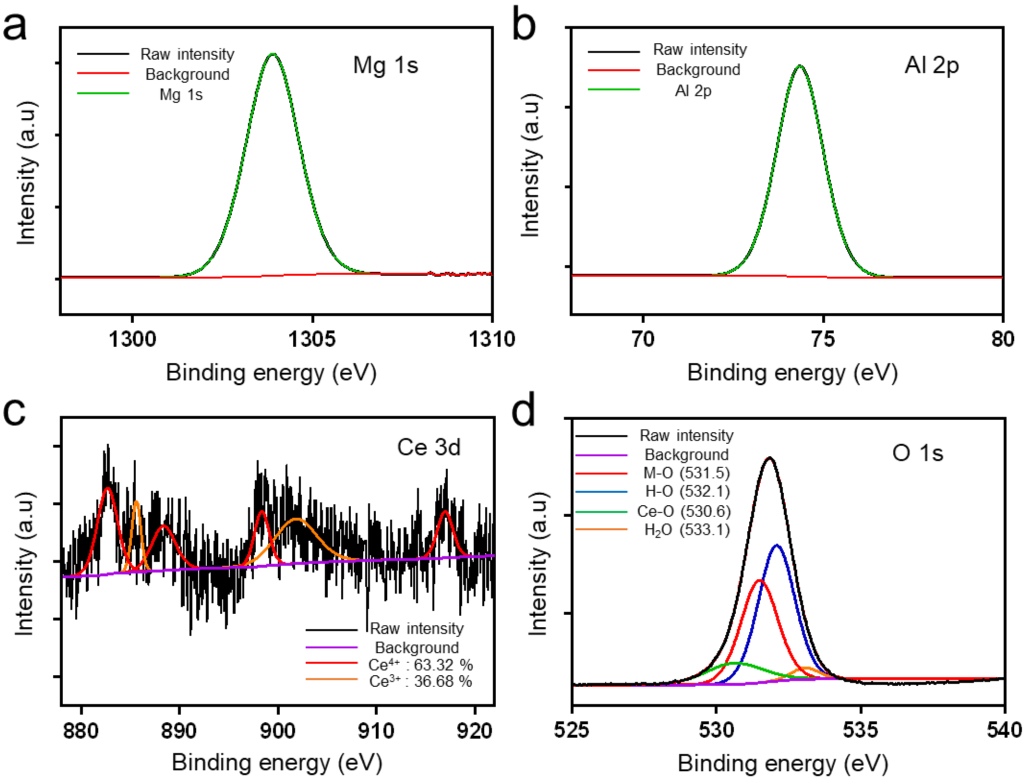

Figure S5. Chemical state of (a) Mg, (b) Al, (c) Ce and (d) O elements in LDH-CeO_2 investigated by XPS.

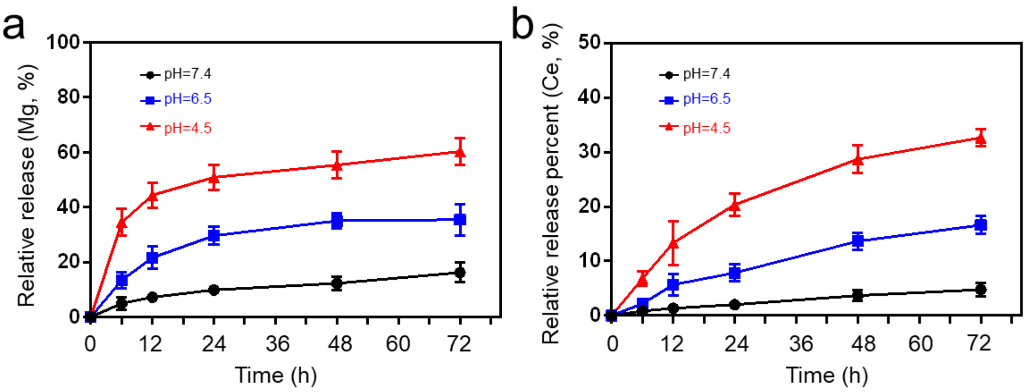

Figure S6. (a) Magnesium ion (Mg^{2+}) and (b) Cerium ($Ce^{3+/4+}$) release profile at PBS solutions (pH = 7.4, 6.5 or 4.5) values.

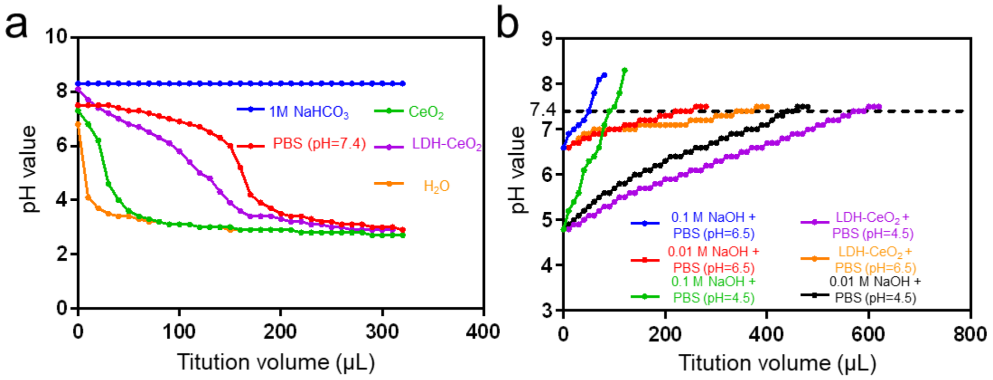

Figure S7. Acid neutralization profile of LDH-CeO_2. (a) pH value variation curves during the HCl acid (1%) titration into LDH-CeO_2, PBS (pH = 7.4), deionized H_2O, CeO_2 NPs, and $NaHCO_3$ (1 M). (b) pH value variation curves during titrations of NaOH (0.1 or 0.01 M), or LDH-CeO_2 into PBS (pH = 4.5 or 6.5).

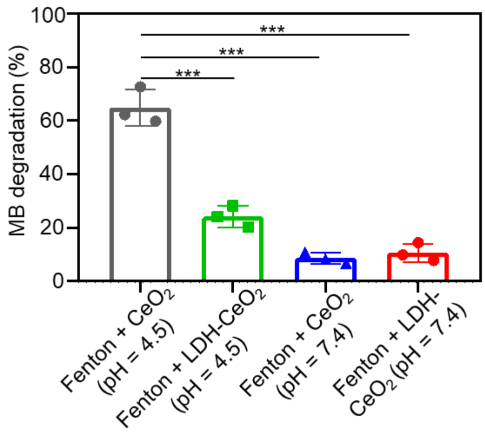

Figure S8. MB decolorization percentage calculated according to absorbance spectra measured by UV-vis. All values are presented as means ± s.d, n= 3. *P < 0.05, **P < 0.01, ***P < 0.001, ns, not significant, two-tailed Student's t test.

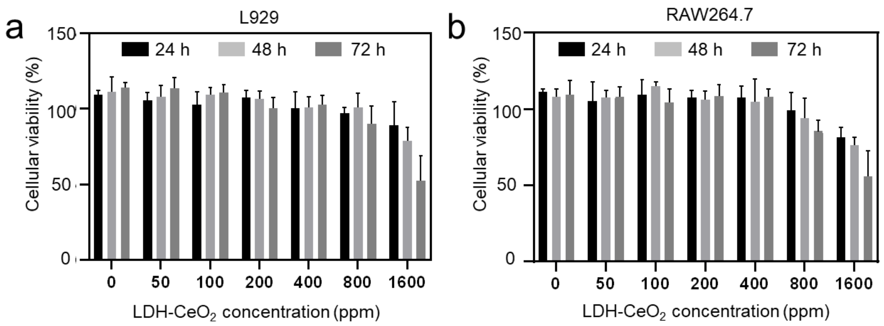

Figure S9. Viabilities of L929 (A) and RAW264.7 (B) cells quantified by CCK-8 assay after incubation with varied concentrations of LDH-CeO2 for 24, 48, and 72 h.

Cell type	Materials	IC50 (ppm, 24 h)	IC50 (ppm, 48 h)	IC50 (ppm, 72 h)
L929	LDH-CeO_2	2233.096	2221.438	1493.308
RAW264.7	LDH-CeO_2	2358.038	2038.037	1572.991

Table S2. The IC50 values of LDH-CeO_2 in L929 and RAW264.7 cell lines.

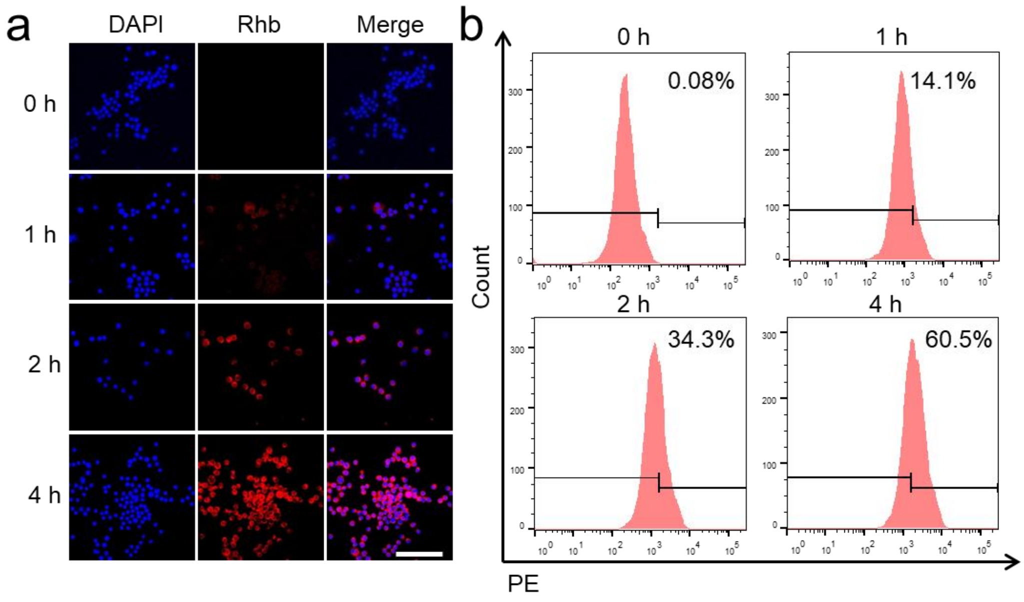

Figure S10. Internalization of LDH-CeO_2 by RAW 264.7 macrophage cell line analysed by (a) CLSM and (b) flow cytometry.

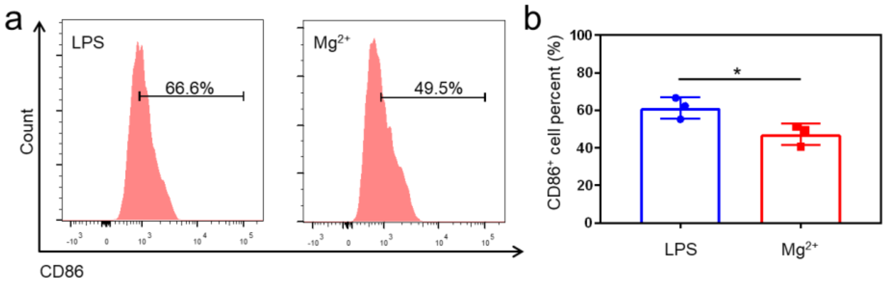

Figure S11. Anti-inflammatory effect of Mg ion in inducing M2 polarization of macrophages. (a) Representative flow cytometry result of CD86 positive cells after treatment by LPS and Mg ion. (b) Corresponding quantification of CD86 positive cells. All values are presented as means ± s.d, n= 3. *P < 0.05, **P < 0.01, ***P < 0.001, ns, not significant, two-tailed Student's t test.

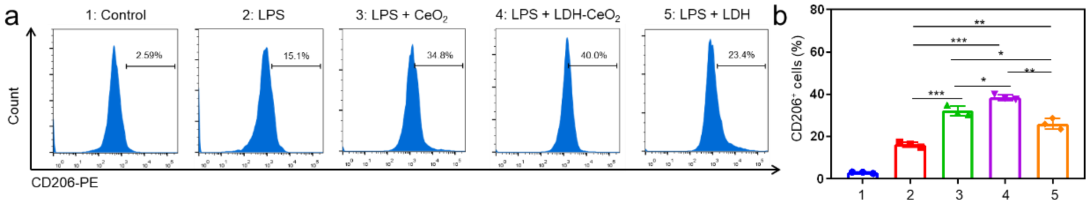

Figure S12. (a) Representative flow cytometry images of CD206 positive cells after

treated by different groups and (b) corresponding quantification. All values are presented as means ± s.d, n= 3. *P < 0.05, **P < 0.01, ***P < 0.001, ns, not significant, two-tailed Student's t test.

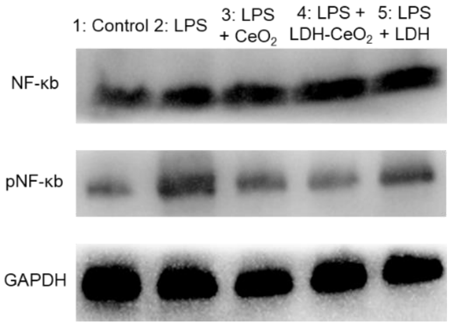

Figure S13. Protein expressions (NF-κB, pNF-κB and GAPDH) examined by the Western Blotting (WB) analyses in macrophages treated by LDH-CeO_2.

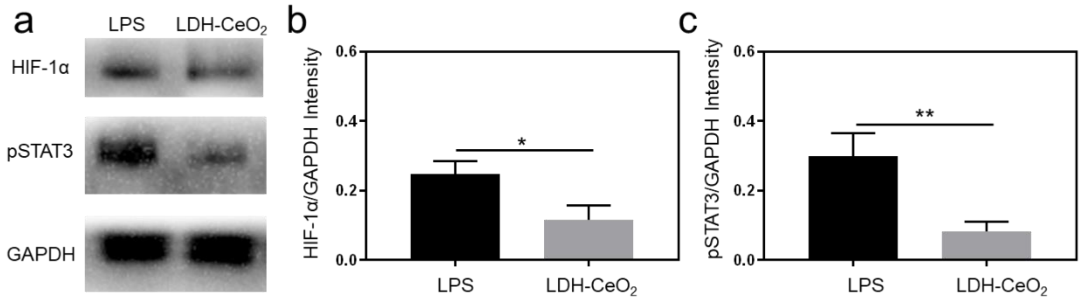

Figure S14. (a) The expression of HIF-1α and pSTAT3 measured by WB assay. Relative intensity quantification of (b) HIF-1α/GAPDH and (b) pSTAT3/GAPDH. All values are presented as means ± s.d, n= 3. *P < 0.05, **P < 0.01, ***P < 0.001, ns, not significant, two-tailed Student's t test.

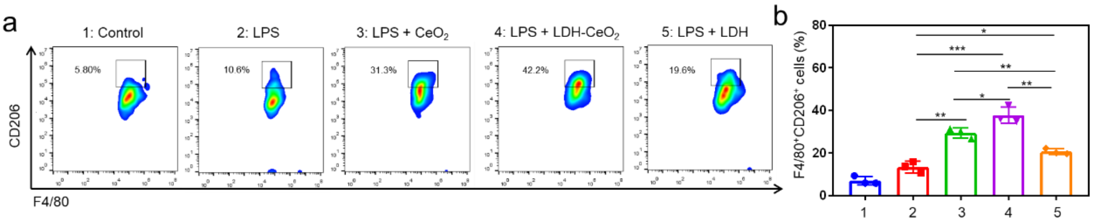

Figure S15. (a) Representative flow cytometry images of F4/80 and CD206 double positive cells after treatments and (b) corresponding quantification. All values are presented as means ± s.d, n= 3. *P < 0.05, **P < 0.01, ***P < 0.001, ns, not significant,

two-tailed Student’s t test.

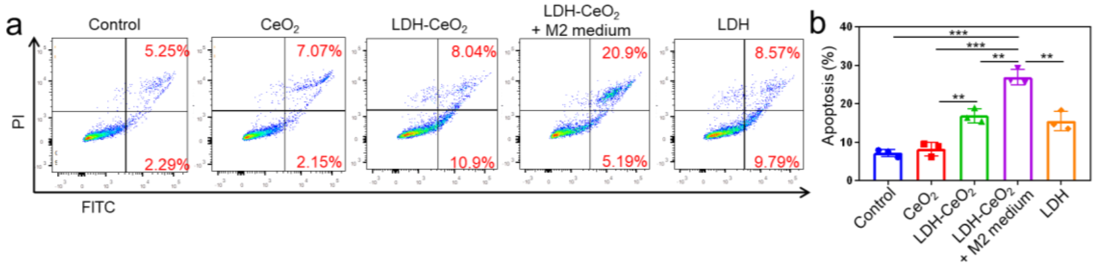

Figure S16. (a) Apoptosis analysis by flow cytometry after treatments and (b) corresponding quantification. All values are presented as means ± s.d, n= 3. *P < 0.05, **P < 0.01, ***P < 0.001, ns, not significant, two-tailed Student’s t test.

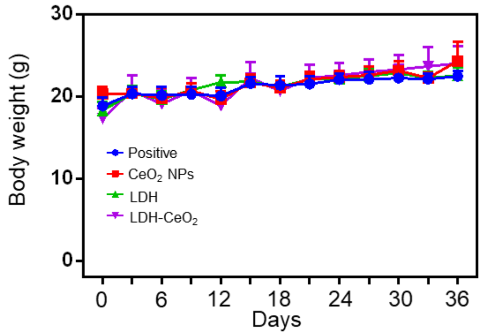

Figure S17. Variation of body weights from RA model mice treated by different groups.

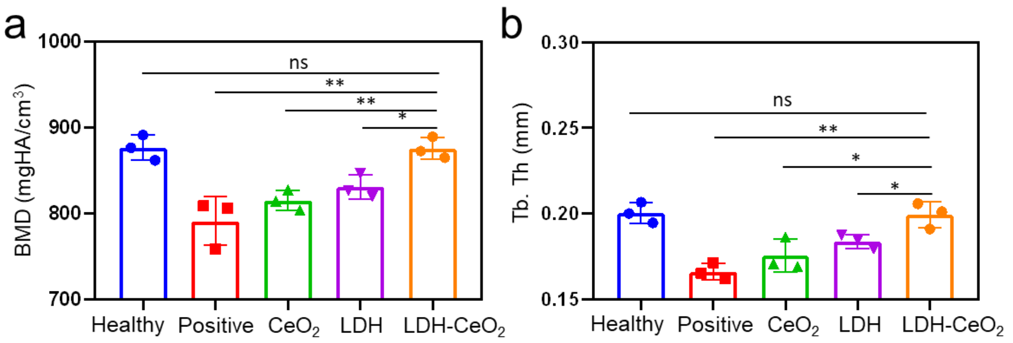

Figure S18. Bone related parameters of (a) BMD (mgHA/cm^3) and (b) Tb. Th (mm) calculated according to micro-CT reconstruction. All values are presented as means ± s.d, n= 3. *P < 0.05, **P < 0.01, ***P < 0.001, ns, not significant, two-tailed Student’s t test.

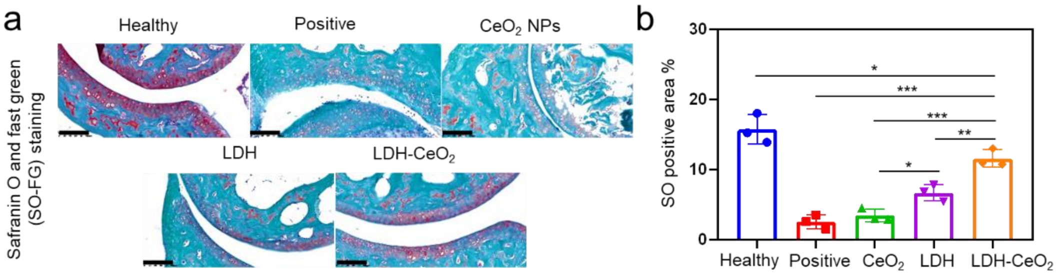

Figure S19. Representative joint sections stained by safranin O and fast green (SO-FG) and corresponding SO positive area quantification. All values are presented as means ± s.d, n= 3. *P < 0.05, **P < 0.01, ***P < 0.001, ns, not significant, two-tailed Student's t test.

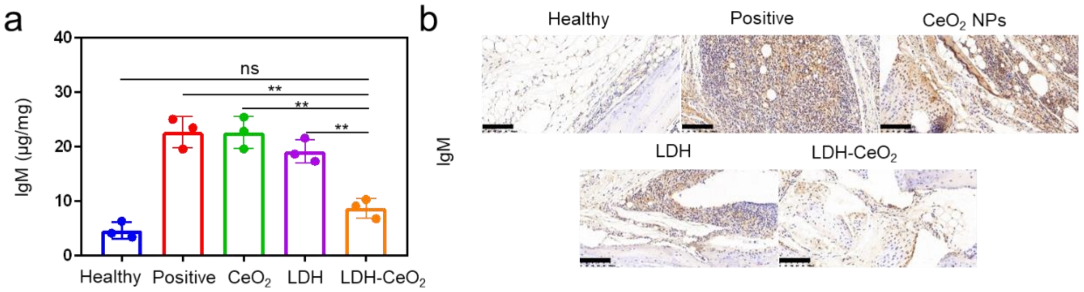

Figure S20. (a) ELISA analysis and (b) immunochemical staining of autoimmune antibody IgM level from joints collected from different groups. All values are presented as means ± s.d, n= 3. *P < 0.05, **P < 0.01, ***P < 0.001, ns, not significant, two-tailed Student's t test.

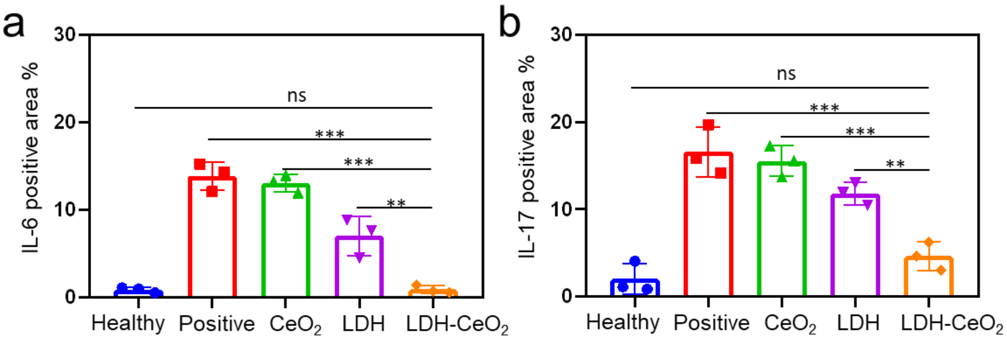

Figure S21. Quantification results of (a) IL-6 and (b) IL-17 positive area from immunochemical staining sections. All values are presented as means ± s.d, n= 3. *P < 0.05, **P < 0.01, ***P < 0.001, ns, not significant, two-tailed Student's t test.

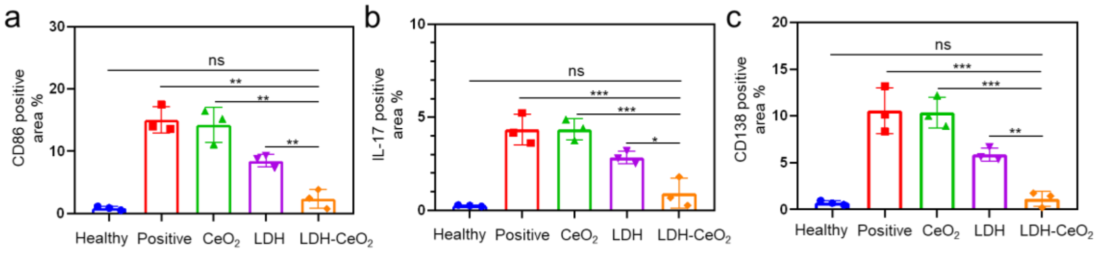

Figure S22. Quantification of (a) CD86, (b) IL-17 and (c) CD138 positive cell percent from immunofluorescence staining sections. All values are presented as means ± s.d, n= 3. *P < 0.05, **P < 0.01, ***P < 0.001, ns, not significant, two-tailed Student's t test.

Group	Total	Dose (mg/kg)	Log Dose	Death	Probits
1	10	300	2.477	0	0.416
2	10	400	2.602	3	2.191
3	10	500	2.699	6	5.722
4	10	600	2.778	8	8.727
5	10	700	2.845	10	9.820

Table S3. Results of the lethal doses of LDH-CeO_2 for determining the LD50 value after injection in mice (n=10).

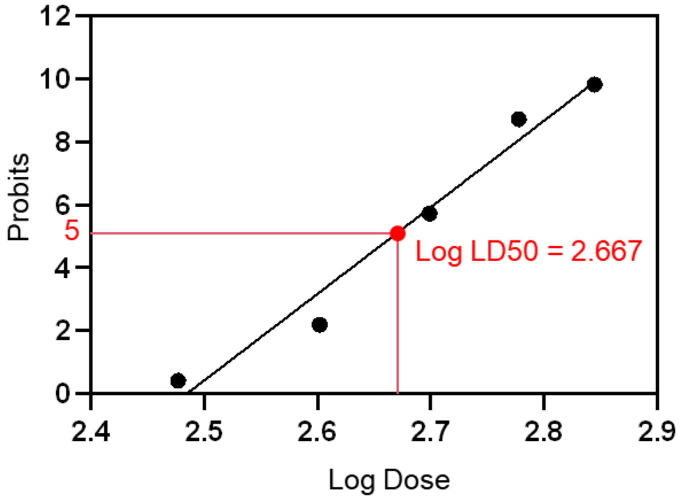

Figure S23. Fitting plot of log-dose versus probits from Table S2 for calculating LD50 value of LDH-CeO_2.

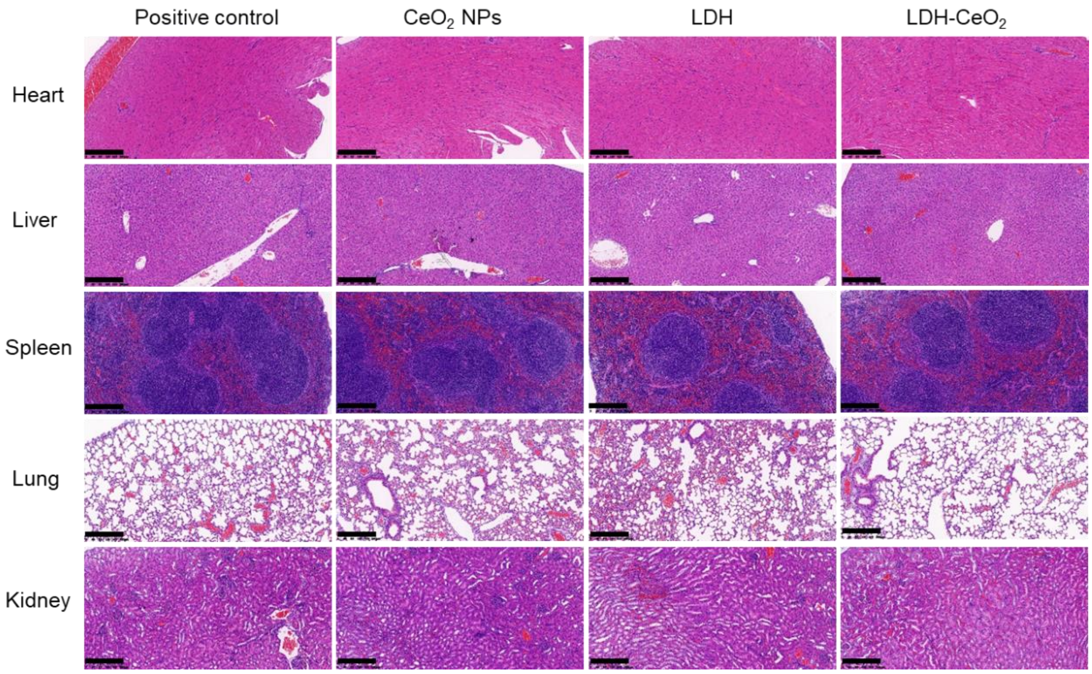

Figure S24. (a) Histological safety evaluation by H&E staining of main organs collected from treated RA mice model. Scale bar: 200 µm.

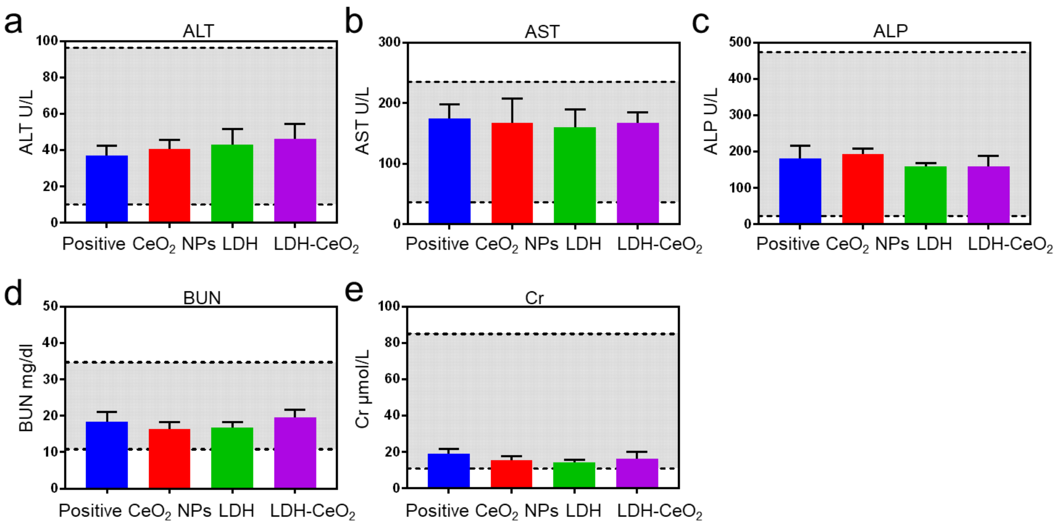

Figure S25. Hematological biocompatibility analyses. Liver functional parameters of (a) ALT, (b) AST and (c) ALP. Kidney functional parameters of (c) BUN and (e) Cr.

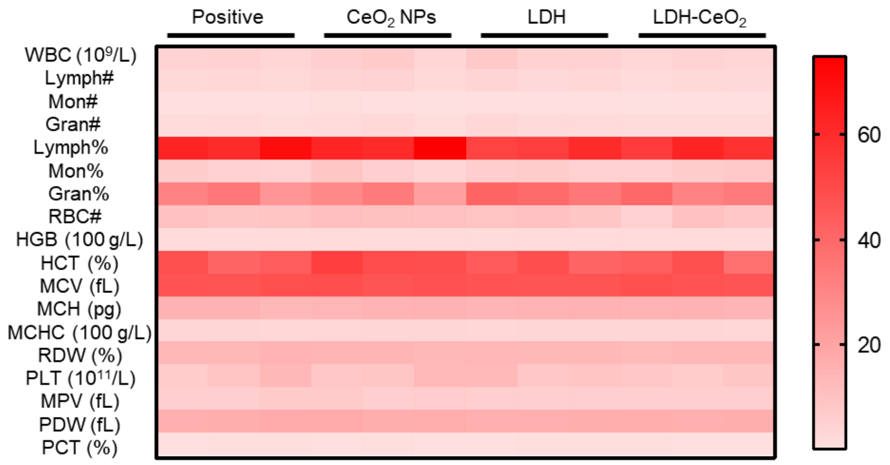

Figure S26. Blood routine indexes examined from mice after the therapeutic period.
